# Dissecting the shared genetic landscape of anxiety, depression, and schizophrenia

**DOI:** 10.1186/s12967-024-05153-3

**Published:** 2024-04-18

**Authors:** Yiming Tao, Rui Zhao, Bin Yang, Jie Han, Yongsheng Li

**Affiliations:** 1 Department of Intensive Care Medicine, Tongji Hospital, Tongji Medical College, Huazhong University of Science and Technology, Hankou, Wuhan, 430030 China; 2 Department of Critical Care Medicine, Shandong Provincial Hospital Affiliated to Shandong First Medical University, Jinan, 250101 Shandong China; 3 Department of Laboratory Medicine, The First Afliated Hospital of Chongqing Medical University, Chongqing, 400042 China; 4 Department of Emergency, School of Medicine, Qingdao Municipal Hospital, Qingdao University, Qingdao, 266071 China

**Keywords:** Anxiety, Depression, Schizophrenia, Shared genetic architecture, Drug targets

## Abstract

**Background:**

Numerous studies highlight the genetic underpinnings of mental disorders comorbidity, particularly in anxiety, depression, and schizophrenia. However, their shared genetic loci are not well understood. Our study employs Mendelian randomization (MR) and colocalization analyses, alongside multi-omics data, to uncover potential genetic targets for these conditions, thereby informing therapeutic and drug development strategies.

**Methods:**

We utilized the Consortium for Linkage Disequilibrium Score Regression (LDSC) and Mendelian Randomization (MR) analysis to investigate genetic correlations among anxiety, depression, and schizophrenia. Utilizing GTEx V8 eQTL and deCODE Genetics pQTL data, we performed a three-step summary-data-based Mendelian randomization (SMR) and protein–protein interaction analysis. This helped assess causal and comorbid loci for these disorders and determine if identified loci share coincidental variations with psychiatric diseases. Additionally, phenome-wide association studies, drug prediction, and molecular docking validated potential drug targets.

**Results:**

We found genetic correlations between anxiety, depression, and schizophrenia, and under a meta-analysis of MR from multiple databases, the causal relationships among these disorders are supported. Based on this, three-step SMR and colocalization analyses identified ITIH3 and CCS as being related to the risk of developing depression, while CTSS and DNPH1 are related to the onset of schizophrenia. BTN3A1, PSMB4, and TIMP4 were identified as comorbidity loci for both disorders. Molecules that could not be determined through colocalization analysis were also presented. Drug prediction and molecular docking showed that some drugs and proteins have good binding affinity and available structural data.

**Conclusions:**

Our study indicates genetic correlations and shared risk loci between anxiety, depression, and schizophrenia. These findings offer insights into the underlying mechanisms of their comorbidities and aid in drug development.

**Supplementary Information:**

The online version contains supplementary material available at 10.1186/s12967-024-05153-3.

## Background

Mental health issues have profound implications for individuals, families, and society, representing a crucial global public health concern [1]. The ongoing COVID-19 pandemic has heightened these concerns, with emerging evidence indicating an increased risk of schizophrenia in severely affected individuals [2]. Post-pandemic, rates of depression and anxiety have surged globally by over 25% [3].

In addition to the direct association with COVID-19, abundant evidence highlights the robust genetic influence on mental disorders, showcasing significant comorbidity and placing these conditions on an age-dependent continuum. The widely accepted p-factor theory proposes a singular dimensional trait that measures individual susceptibility to mental disorders, comorbidity, disease duration, and symptom severity [4]. Supported by evidence across symptomatology, pathology, and genetics, this theory forms a crucial foundation for our study.

The complex comorbidity of diverse mental disorders not only complicates treatment but also substantially affects patients' quality of life and daily functioning [5]. Although ample evidence points to a robust genetic basis for the high comorbidity of mental disorders, the complex etiology often makes it challenging to attribute the occurrence of mental disorders to a single genomic location [6]. This gap in understanding presents a significant challenge in effectively guiding the treatment of mental disorders and drug development. However, Genome-Wide Association Studies (GWAS) have successfully linked hundreds of specific genetic loci to mental disorders, offering the potential to alter this situation [7].

Mendelian Randomization (MR) offers an experimentally designed approach, utilizing the natural distribution of genetic variations revealed by GWAS to tackle causal inference challenges in observational studies on mental disorders [8]. Additionally, LD Score regression (LDSC), a statistical method utilizing GWAS data, enables the evaluation of genetic correlation among different loci, providing an estimate of the genetic relatedness among phenotypes [9]. In this study, we conduct a comprehensive analysis of the genetic correlation and causal associations among anxiety, depression, and schizophrenia using data from multiple GWAS sources, employing MR and LDSC.

While GWAS excels in identifying SNP variations associated with mental disorder risks, it falls short in pinpointing the exact causative genes, posing challenges to direct drug development [10, 11]. The Summary-data-based Mendelian Randomization (SMR) method aims to investigate pleiotropic associations between gene expression levels and specific complex traits, using summarized data from GWAS and expression quantitative trait loci (eQTL) studies [12]. In this study, extending the exploration of genetic correlation among three mental disorders, we employ the SMR technique to reveal core genes and proteins that may play functional roles in anxiety, depression, and schizophrenia. This promises a more precise understanding of the diseases' pathogenesis and enables an analysis of shared risk loci.

**Fig. 1:**
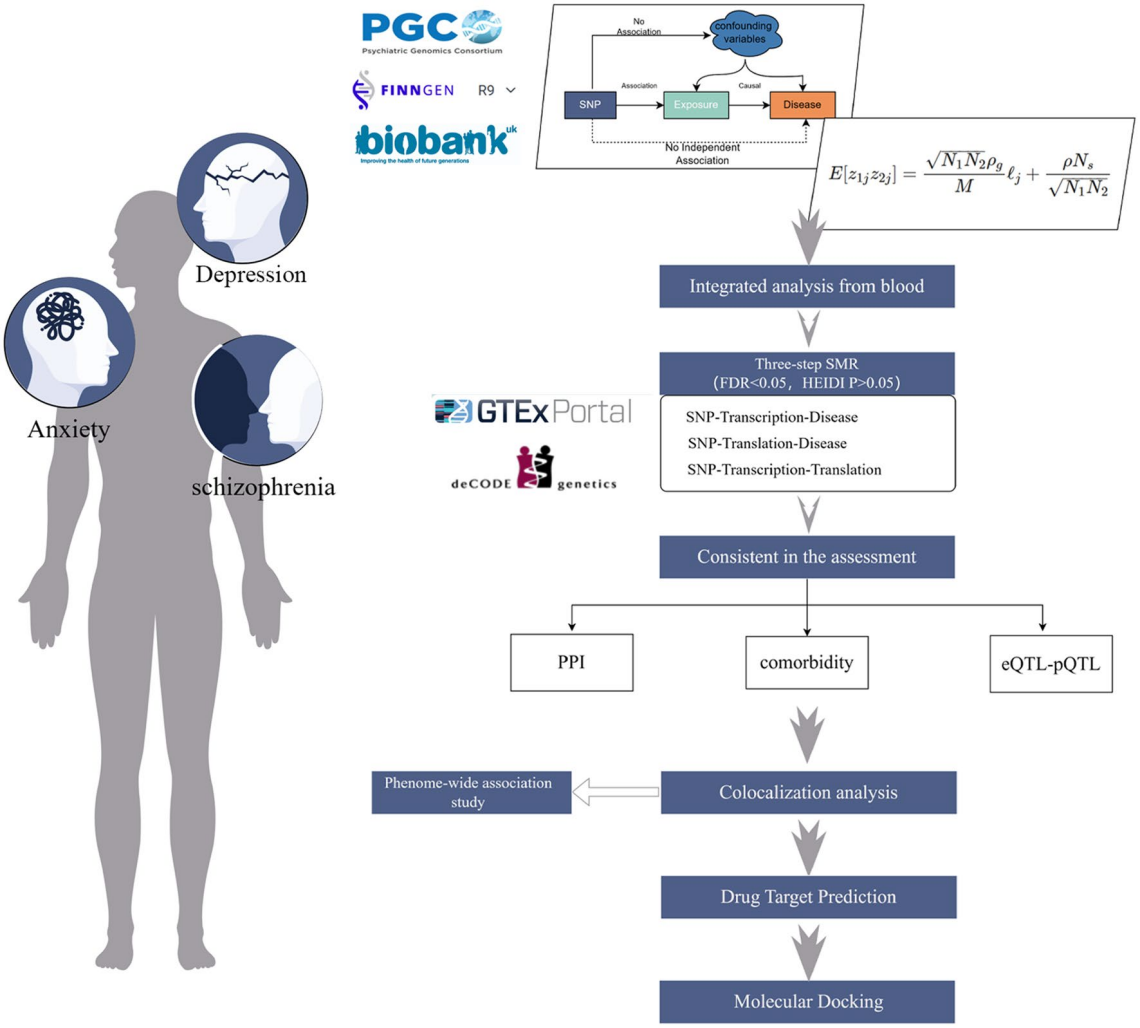
Study design. SNP, single nucleotide polymorphism; SMR, summary-data-based mendelian randomization; PPI, Protein–Protein Interaction

Subsequently, through a colocalization analysis of GWAS and QTL based on SMR findings, we confirm potential therapeutic targets and common driving factors between therapeutic targets and the risk of mental disorders. This analysis aids in determining the causal relationship between therapeutic targets and diseases while eliminating potential confounding factors [13]. Building upon this foundation, phenome-wide association studies (PheWAS) analysis explores associations between potential therapeutic targets and other features, offering insights into their multifunctionality and potential impact mechanisms for further research and development of related therapeutic strategies [14]. Finally, by consolidating results from multiple drug target databases, molecular docking studies are conducted to discover and validate the credibility of therapeutic targets at the atomic level through computer simulation techniques [15]. The study design is presented in Fig. 1.

In conclusion, our research endeavors to unravel the pathogenesis of anxiety, depression, and schizophrenia individually, exploring their genetic correlation, causal relationships, and shared risk loci with potential functional roles. By integrating results from MR, LDSC, SMR, co-localization analysis, and PheWAS, we aim to provide valuable insights for the development of more effective and targeted treatment methods, thereby addressing a critical gap in current mental health research.

## Methods

### Datasets

#### GWAS summary statistics

We obtained GWAS data for anxiety, depression, and schizophrenia from three databases: Finngen R9, UK Biobank (UKBB), and the Psychiatric Genomics Consortium (PGC). These databases collectively provide comprehensive genetic insights into psychiatric disorders. The Finngen database, with a focus on the Finnish population, contains genetic data from hundreds of thousands of individuals and covers various clinical indicators related to psychiatric disorders [16]. UKBB, a large-scale biobank project in the United Kingdom, encompasses genetic, clinical, and lifestyle data from approximately 500,000 participants, offering a vast dataset of genetic markers associated with psychiatric conditions [17]. The PGC database is an international collaborative initiative that consolidates genetic data from diverse regions, facilitating large-scale cross-population studies on psychiatric disorders [18–21]. Detailed database links and references can be found in Additional file 2: Table S1.

#### eQTL summary statistics

Expression Quantitative Trait Loci (eQTL) are genetic variations or single nucleotide polymorphism (SNP) loci that influence gene expression levels. These loci exhibit associations with specific genes' expression levels in an individual's genome [22]. Studying eQTL provides insights into genetic factors that regulate gene expression, shedding light on gene functionality and its links to phenotypes. The Genotype-Tissue Expression (GTEx) project is a vital resource that collects tissue samples from diverse healthy individuals, encompassing organs like the heart, liver, kidney, lungs, brain, and more [23]. With contributions from thousands of donors, GTEx offers extensive eQTL data, revealing connections between genotypes and gene expression levels. In our analysis, we focused on GTEx V8 whole blood cis-eQTL summary statistics (p < 1 × 10^–5^) for SMR analysis.

#### pQTL summary statistics

Protein Quantitative Trait Loci (pQTL) are genetic variations or loci linked to changes in protein levels [24]. Like eQTL, pQTL represents genomic positions associated with specific protein expression levels. These loci can co-localize with disease variants, helping identify pathogenic proteins, disease pathways, and potential drug targets. The deCODE Genetics team conducted a large-scale Genome-Wide Association Study (GWAS) using plasma proteomics technology. This study involved 35559 individuals from Iceland, analyzing 4907 proteins and identifying 18084 pQTLs. These pQTLs establish connections between plasma protein levels and 373 diseases and other traits based on genetic variations [25]. For our dataset, we initially filtered using a threshold of 1 × 10^–5^. Subsequently, we conducted local plink clumping with default conditions (R^2^ = 0.001, kb = 10000) using the 1000 Genomes European Phase V3 file as the reference. We then prepared the necessary files in the required format for SMR analysis according to SMR analysis guidelines.Included studies had been approved by corresponding ethical review committees.

### Mendelian randomization

#### Selection of genetic instruments

To explore potential causal relationships among the three psychiatric disorders, we conducted MR analysis using the R package "TwoSampleMR." Following the principles of the Instrument-Relevance Assumption, Instrument-Independence Assumption, and Exclusion Restriction Assumption, we employed strict criteria (P < 5 × 10^–8^) to select SNPs related to the exposure factor. For SNPs sourced from the anxiety phenotype GWAS data in the PGC database, we adjusted the screening threshold to P < 5 × 10^–6^ to ensure an adequate number of SNPs for analysis.

Cluster analysis utilized a window size of 10,000 kb and a threshold of R^2^ < 0.001. To minimize potential biases, we harmonized exposure and outcome variables, ensuring consistent matching of effect alleles within the same allele gene [26]. For each instrumental variable (IV), we systematically searched the PhenoScanner GWAS database, excluding any SNPs associated with confounding factors to mitigate potential bias [27].

#### Statistical analysis

The primary method employed in the MR analysis is the Inverse Variance Weighted (IVW) method, which effectively addresses heterogeneity among genetic instruments, enhancing the accuracy of estimates [28]. In addition to the IVW method, we employed various other MR models, including Weighted Median, MR-Egger, Simple Model, and Weighted Mode, to validate causal relationships [29]. To assess heterogeneity in gene exposure-outcome associations, Cochran’s Q test was utilized, comparing the variability in the estimation of genetic variant effects [30]. To account for potential outliers and pleiotropy, we applied the MR-Presso method for outlier correction [31]. For the assessment of horizontal pleiotropy, the MR-Egger intercept test was employed, using regression to evaluate the impact of genetic variation on exposure effect-associated outcomes, with p < 0.05 indicating the presence of horizontal pleiotropy [32].

For each SNP, a leave-one-out analysis was conducted, and a forest plot was generated to assess their individual contributions. Finally, the MR analysis results from multiple sources of GWAS data were subjected to meta-analysis to provide robust evidence for causal inference.

### LDSC analysis

Genetic correlation refers to the correlation generated by genotypes among phenotypes in a population. In GWAS analyses, the estimated effect size for a SNP often includes the effects of other SNPs in linkage disequilibrium (LD) with that SNP, meaning that SNPs with higher LD tend to have higher chi-square test statistics. This fact remains true when we replace the chi-square test statistic with the product of Z-scores from GWAS of two correlated phenotypes^9^. Based on this principle, LDSC can be utilized to estimate the heritability of a trait and the genetic correlation across traits from GWAS summary statistics [33].

In our study, the “ldscr” R package was employed for LD score regression. The 1000 Genomes Phase 3 data of European ancestry served as the reference panel for calculating LD scores, and only SNPs in HapMap 3 with a minor allele frequency (MAF) > 0.05 were included as input [34]. In the results, *rg* represents the genetic correlation between two traits, ranging from -1 to 1; values closer to 1 or -1 indicate stronger correlation. r_g__P denotes the statistical significance, with values below 0.05 considered statistically significant.

### Three-step SMR analysis

Based on the 1000 Genomes European reference, and utilizing SMR analysis, we conducted a three-step analysis to determine causal inferences between genetic loci and three mental disorders: (1) SMR analysis of GWAS data for three mental disorders and eQTL data from GTEx V8; (2) SMR analysis of GWAS data for three mental disorders and pQTL data from deCODE Genetics; (3) SMR analysis of eQTL data from GTEx V8 and pQTL data from deCODE Genetics, with a focus on important signals identified in steps 1 and 2.

As a default, SNPs in strong linkage disequilibrium (LD) with an R^2^ > 0.9 were removed, along with those associated with top eQTLs if the minor allele frequency (MAF) was > 0.01. Significant SMR probes were selected based on false discovery rate (FDR)-corrected thresholds for SMR P values < 0.05, and HEIDI test P value thresholds > 0.05 were applied to indicate the lack of heterogeneity [12].

The SMR analysis was performed on GWAS data from all three database sources (Finngen, UKBB, PGC), as described above. The selected genes and protein loci must meet the filtering criteria in at least one database and maintain consistent directionality across all databases. In other words, the impact trend of these loci on the diseases should be consistent across all databases.

### Analysis and screening of loci

The selected loci identified through SMR analysis were subjected to three analysis methods: (1) Protein–Protein Interaction (PPI) Analysis: Identifying key loci through the analysis of protein–protein interactions; (2) Cross-Analysis of Causal Loci for anxiety, depression, and schizophrenia: exploring comorbid loci by analyzing loci implicated in the causation of anxiety, depression, and schizophrenia; (3)Selection of Implicated Loci in all three mental disorders: Selecting signals that meet the criteria of FDR < 0.05 and HEIDI_P > 0.05 in all three steps of SMR analysis. These are indicative loci that exert an influence on all three disorders at both the genetic and protein levels.

#### PPI analysis

Having conducted PPI analysis on the loci identified through SMR analysis for each of the three disorders, the underlying principle of PPI involves physical or chemical interactions between different proteins, leading to structural changes that can impact their functionality [35]. The online STRING database (string-db.org) was employed for PPI analysis with a medium confidence score set at 0.4.

To validate the hub genes, topological analysis methods, specifically the degree algorithm, were applied. The CytoHubba plugin, integrated into Cytoscape 3.8.0 (University of California, San Diego, CA, USA), was utilized for this purpose [36]. Through an analysis of the topological structure of the PPI network, key nodes were identified.

#### Comorbidity locus analysis

A cross-analysis was performed on the pathogenic loci identified by SMR analysis for the three disorders, aiming to identify common loci and determine whether these loci exhibit consistent directional effects (either as risk factors or protective factors) across all diseases.

#### Three-step SMR analysis

Screening for anxiety, depression, and schizophrenia identified loci that met the selection criteria in the Three-step SMR analysis. These loci provide systematic evidence for correlations: the correlation between gene expression and disease, the correlation between corresponding protein expression and disease, and the impact of gene expression on corresponding protein expression levels. This analysis substantially enhances the reliability of the identified loci.

### Colocalization analysis

Colocalization analysis was conducted for the loci identified in anxiety, depression, and schizophrenia using GWAS and QTL data. This analysis aimed to confirm potential therapeutic targets and identify common driving factors associated with the risk of the three mental disorders. Its purpose was to strengthen the evidence of the association between targets and disease phenotypes, facilitating the determination of causal relationships between therapeutic targets and diseases while minimizing potential confounding factors [37, 38].

The colocalization analysis was conducted using the “coloc” R package, with a threshold of PPH4 > 0.8. This threshold was used to determine the presence of shared genetic effects between the targets and phenotypes, providing a reliable basis for further investigation and therapeutic development [39, 40].

### PheWAS analysis

We performed a PheWAS analysis on GWAS summary statistics resources using the GWAS ATLAS analysis tool [41–43]. This analysis enabled us to investigate the associations of individual genetic loci with a wide range of traits across various GWAS datasets. We systematically examined and summarized the loci identified in the three mental disorders, shedding light on the multifunctionality of these loci and the potential mechanisms underlying their effects on various traits [44].

### Candidate drug prediction

Before delving into further research and development of potential therapeutic drugs, we explored existing practical drugs' interactions with the identified targets by assessing protein-drug interactions [45]. Specifically, we utilized five drug target prediction databases: DrugBank [46], Therapeutic Target Database [47], ChEMBL [48], DGIdb [49], and PharmSnap. These databases link drugs and other chemical substances to their target genes, aiding in the identification and prediction of candidate drugs targeting the discovered loci in our study.

This comprehensive approach enhances our understanding of the potential therapeutic interventions for the studied mental disorders and paves the way for the development of novel treatment strategies.

### Molecular docking analysis

To gain deeper insights into the impact of candidate drugs on the drug targets associated with the three mental disorders and assess the druggability of these targets at the atomic level, our study conducted molecular docking analysis. We employed Autodock Vina 1.2.2, a computational protein–ligand docking software, to evaluate the binding energy and interaction patterns between candidate drugs/small molecules and their respective targets [50].

The drug structure data were obtained from the PubChem compound database, while the protein 3D structure data were sourced from the PDB database [51]. All protein and molecule files were converted into PDBQT format, with the exclusion of water molecules and the addition of polar hydrogen atoms. The grid box dimensions were configured to 30 Å × 30 Å × 30 Å, with a grid point distance of 0.05 nm. Molecular docking studies were carried out using Autodock Vina 1.2.2.

## Results

### Results of Mendelian randomization analysis

**Fig. 2:**
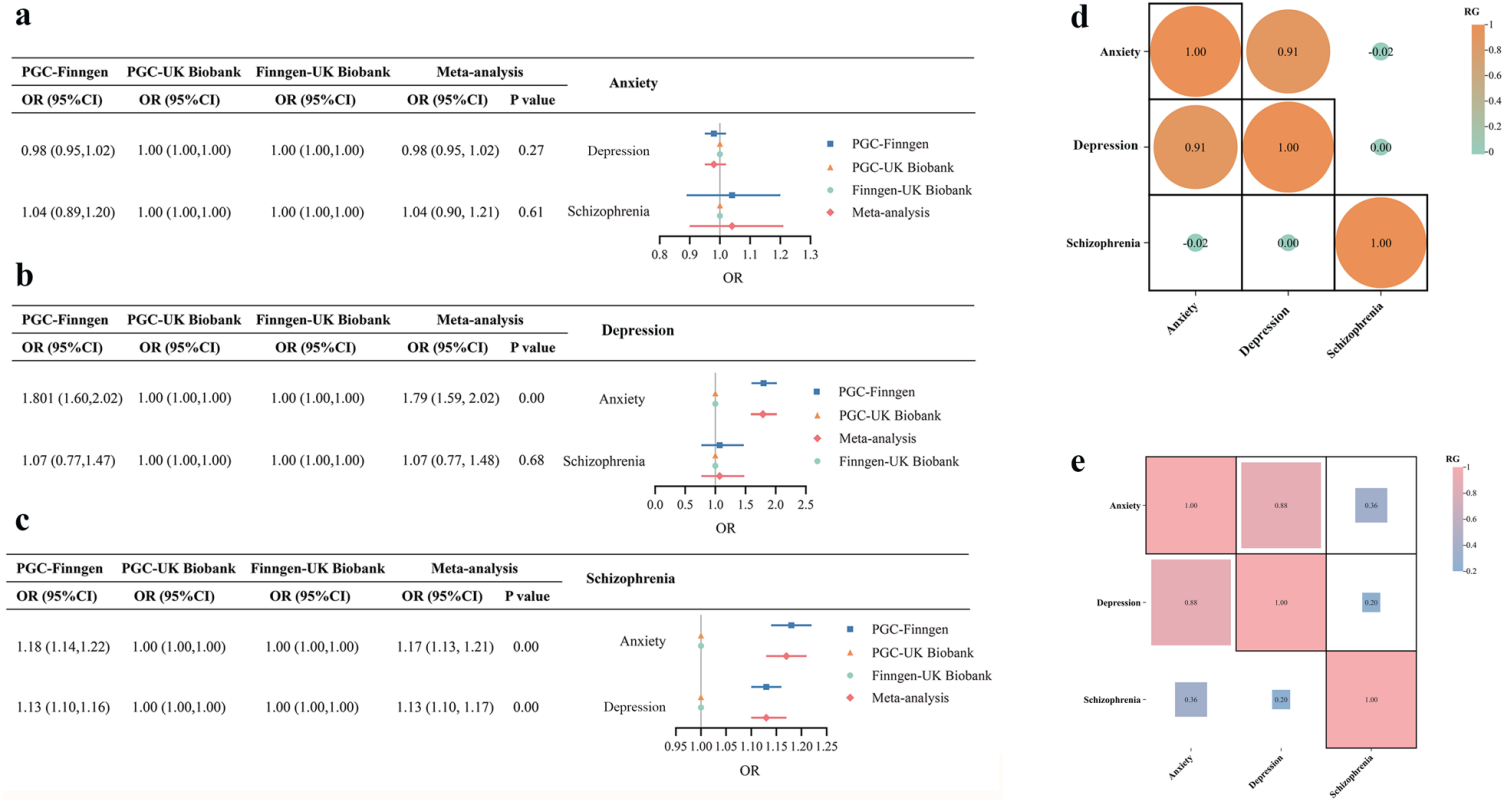
Summary of MR and LDSC analysis results between anxiety disorder, depression, and schizophrenia. **a** Results of MR Analysis of anxiety and other two diseases; **b** Results of MR Analysis of depression and other two diseases; **c** Results of MR Analysis of schizophrenia and other two diseases; **d** LDSC analysis using GWAS data from PGC database; **e** LDSC analysis using GWAS data from Finngen database.MR, Mendelian randomization; LDSC, linkage disequilibrium score regression

We conducted bidirectional MR analysis using data from three databases to explore causal relationships among three mental disorders. Specifically, we used Finngen-derived GWAS data as the exposure variable with UKBB data as the outcome, PGC-derived GWAS data as the exposure variable with Finngen data as the outcome, and PGC-derived GWAS data as the exposure variable with UKBB data as the outcome. Utilizing five different MR methods and performing meta-analysis, we identified evidence supporting causal relationships between depression and anxiety (IVW OR = 1.79; 95% CI 1.59–2.02; P < 0.05), schizophrenia and anxiety (IVW OR = 1.17; 95% CI 1.13–1.21; P < 0.05), as well as schizophrenia and depression (IVW OR = 1.13; 95% CI 1.10–1.17; P < 0.05) (Fig. 2a–c and Additional file 2: Table S2). The analysis showed no significant heterogeneity or evidence of pleiotropy. Leave-one-out analysis indicated that the results were not driven by any single SNP (Additional file 1: Figs. S1–S18). Overall, this analysis provides strong evidence for the existence of causal relationships among these mental disorders, highlighting their intricate genetic connections.

### Estimation of genetic correlations

We employed LDSC analysis to assess the genetic correlations among anxiety disorder, depressive disorder, and schizophrenia. Analysis using GWAS data from the PGC revealed the following correlation estimates: between anxiety and depressive disorder (r_g_ = 0.907, P = 4.44 × 10–21), between anxiety and schizophrenia (r_g_ = − 0.0175, P = 0.472), and between depressive disorder and schizophrenia (r_g_ = 0.00121, P = 0.863) (Fig. 2d). Analysis using GWAS data from the FinnGen database yielded similar correlation estimates: between anxiety and depressive disorder (r_g_ = 0.884, P = 2.64 × 10^–39^), between anxiety and schizophrenia (r_g_ = 0.356, P = 3.34 × 10^–5^), and between depressive disorder and schizophrenia (r_g_ = 0.201, P = 0.00494) (Fig. 2e). These results indicate a strong and consistent genetic correlation between anxiety and depressive disorder (Additional file 2: Table S3). In the FinnGen database, there is also a moderate genetic correlation between anxiety and schizophrenia, as well as between depressive disorder and schizophrenia, although it is relatively weaker. Furthermore, in the PGC database, this genetic correlation is weak and less stable.

### Integration of GWAS and mental disorders-related QTL data from three databases

To identify potential pathogenic genes for the three mental disorders and explore their genetic mechanisms in gene regulation and protein translation, we conducted a Three-step SMR Analysis. Significant results from each step were systematically documented as indicative causal genes or proteins (Additional file 1: Figs. S19–S30). Within the three databases, we identified a total of 5 cis-eQTLs that met the criteria and exhibited suggestive causal relationships with anxiety, 39 with depression, and 217 with schizophrenia. Notably, 2 cis-eQTLs consistently affected Anxiety, 27 affected Depression, and 97 influenced Schizophrenia across all three databases (Additional file 2: Table S4).

Furthermore, for cis-pQTLs that met the filtering criteria, 2 displayed suggestive causal relationships with Anxiety, 12 with Depression, and 41 with Schizophrenia. Among these, 1 cis-pQTL for Anxiety, 11 for Depression, and 17 for Schizophrenia consistently influenced all three disorders in the same direction across the three databases (Additional file 2: Table S5).

**Fig. 3:**
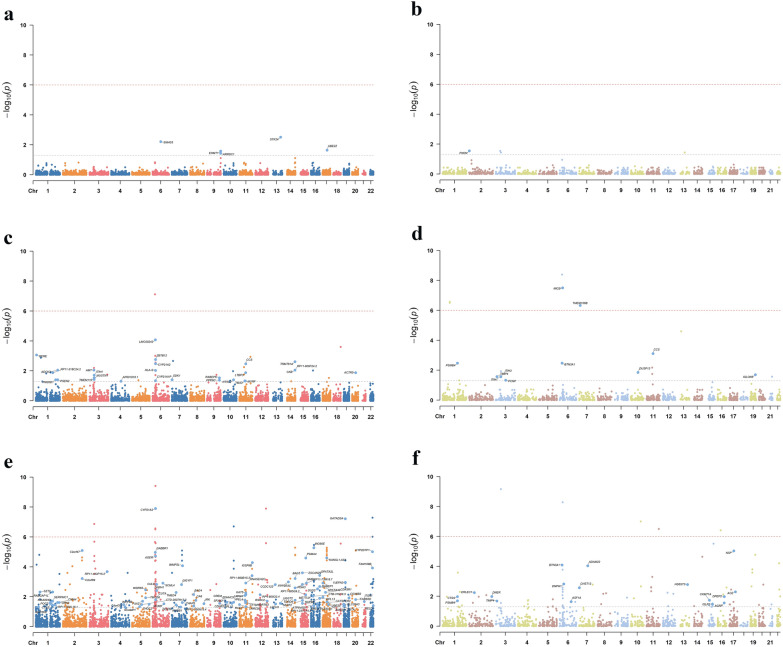
Manhattan plots depicting the use of SMR to screen for QTLs in whole blood related to anxiety, depression, and schizophrenia. **a** Manhattan plots depicting eQTLs in whole blood related to anxiety; **b** Manhattan plots depicting pQTLs in whole blood related to anxiety; **c** Manhattan plots depicting eQTLs in whole blood related to depression; **d** Manhattan plots depicting pQTLs in whole blood related to depression; **e** Manhattan plots depicting eQTLs in whole blood related to schizophrenia; **f** Manhattan plots depicting pQTLs in whole blood related to schizophrenia. SMR, Summary-data-based Mendelian Randomization; eQTL, expression quantitative trait loci; pQTL, Protein Quantitative Trait Loci. Chr, Chromosome

To enhance understanding of the genetic characteristics of these mental disorders, we created manhattan plots to visually illustrate the genotype distribution of key eQTLs and pQTLs associated with anxiety, depression, and schizophrenia at both the genetic and protein regulation levels (Fig. 3).

### Core site analysis and verification

After identifying genes and protein sites that may be causally related to anxiety, depression, and schizophrenia, as described in the methods section, we proceeded with three analytical methods for further validation.

#### PPI Analysis

**Fig. 4:**
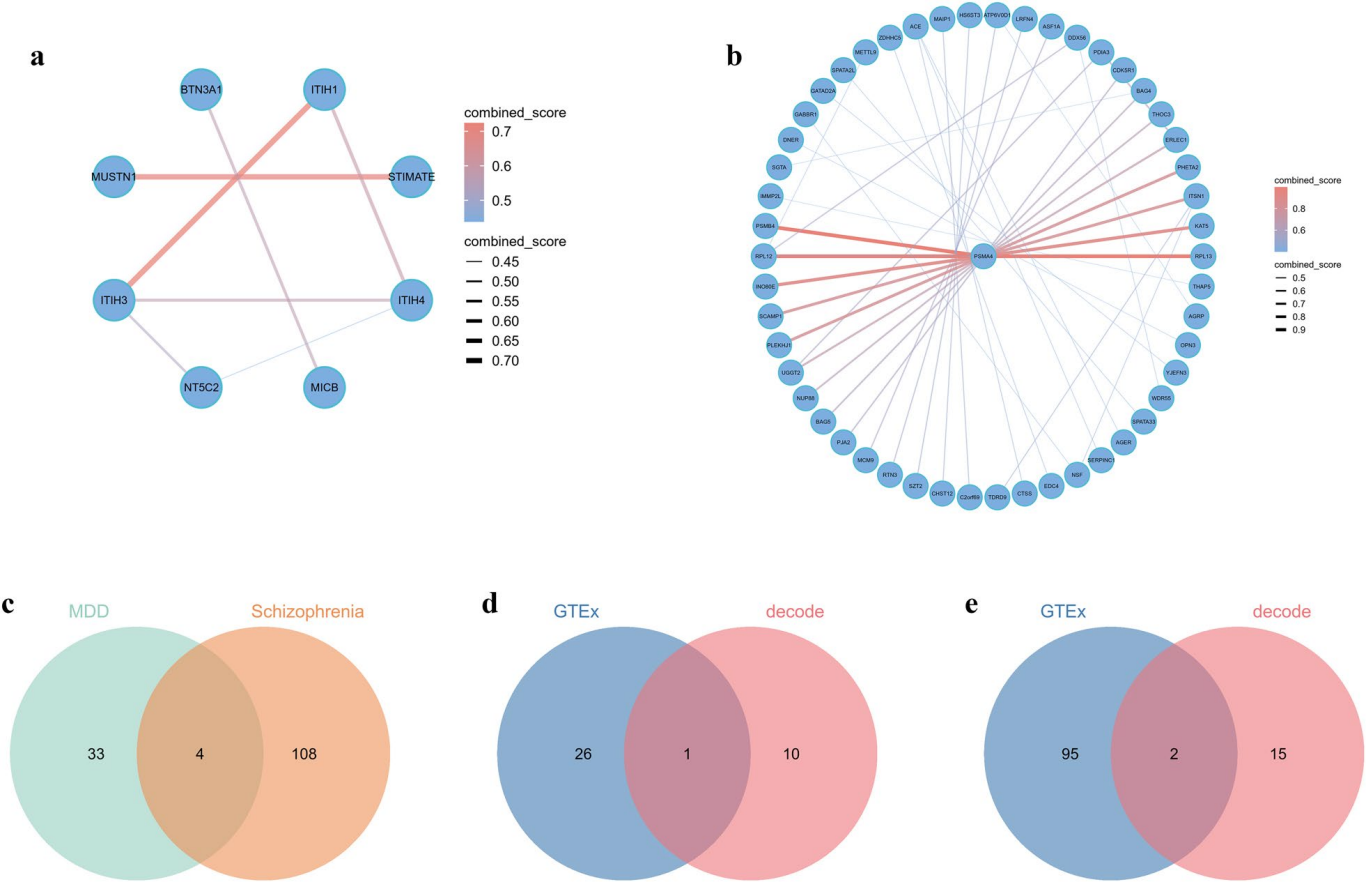
Core Site Analysis and Verification. **a** PPI analysis on the loci identified through SMR analysis for depression; **b** PPI analysis on the loci identified through SMR analysis for schizophrenia; **c** comorbidity sites between depression and schizophrenia; **d** Three-step SMR Analysis sites for depression; **e** Three-step SMR Analysis sites for schizophrenia. PPI, Protein–Protein Interaction; SMR, Summary-data-based Mendelian Randomization

Due to the limited number of anxiety-related sites, constructing a PPI network was challenging. Consequently, we performed PPI network analysis on pathogenic sites associated with depression and schizophrenia. Using Cytoscape software, we identified and visualized the relationships within these subnetworks. The biological significance of proteins within these subnetworks was assessed by examining depression and schizophrenia betweenness centrality. This metric identifies 'bottleneck' nodes crucial for communication within the network. Notably, ITIH3, ITIH4, and NT5C2 were selected for depression (Fig. 4a), while PSMA4 and ITSN1 were identified for schizophrenia (Fig. 4b).

#### Comorbidity site analysis

We did not identify any comorbidity sites between anxiety and depression or schizophrenia. However, we conducted a cross-analysis of potential sites associated with depression and schizophrenia, which revealed four intersecting sites: BTN3A1, CYP21A2, PSMB4, and TIMP4 (Fig. 4c). The consistent impact of these four sites on both depression and schizophrenia suggests that they may be shared factors contributing to the comorbidity of these two disorders.

#### Three-step SMR analysis-identified site

We filtered sites that met the criteria in all three steps of the Three-step SMR analysis for anxiety, depression, and schizophrenia. These sites satisfy both the correlation between gene expression and the disease and the correlation between corresponding protein expression and the disease, concurrently demonstrating that the gene expression level can impact the corresponding protein expression level. A total of three such sites were identified: CCS for depression and CTSS and DNPH1 for schizophrenia (Fig. 4d, e).

### Colocalization verification

To verify the co-localization of the sites identified in the screening of anxiety, depression, and schizophrenia, we conducted co-localization analysis. We performed co-localization analysis on PPI core sites, comorbidity sites, and sites filtered through the three-step SMR analysis. The co-localization results, with PPH4 > 0.8, included ITIH3, BTN3A1, PSMB4, TIMP4, CCS, CTSS, and DNPH1 from the three-step SMR analysis. These results are presented in Table 1.

**Table 1:** Colocalization results of QTLs for site with mental disorder

*PPI*			
Target	Mental disorders	eQTL-PP.H4	pQTL- PP.H4
ITIH3	Depression		0.999
ITIH4	Depression	0.069	
NT5C2	Depression	0.000	
ITSN1	Schizophrenia	0.003	
PSMA4	Schizophrenia	0.000	

Comorbidity sit			
Target		depression-PP.H4	schizophrenia-PP.H4
CYP21A2	Depression/schizophrenia	0.000	0.000
BTN3A1	Depression/schizophrenia	0.969	0.969
PSMB4	Depression/schizophrenia	1.000	1.000
TIMP4	Depression/schizophrenia	0.992	0.992

Three-step SMR analysis			
Target		eQTL-PP.H4	pQTL-PP.H4
CCS	Depression	0.000	0.989
CTSS	Schizophrenia	0.013	0.998
DNPH1	Schizophrenia	0.000	0.998

### Identification of key genes and proteins

Through our analysis, we identified several key genes and proteins associated with the onset of anxiety, depression, and schizophrenia. Notably, sites such as ITIH3, CCS, CTSS, and DNPH1 were found to play pivotal roles in shaping the genetic landscape of mental health. ITIH3 is involved in regulating inflammation and neurotransmitter balance, while CCS is implicated in the regulation of oxidative stress and copper homeostasis. Additionally, tissue proteinase S (CTSS) is renowned for its involvement in protein degradation and maintenance of blood–brain barrier integrity, suggesting potential connections with schizophrenia. Furthermore, DNPH1, which is involved in axonal transport and brain structure, may be associated with the development of schizophrenia. These findings align closely with our current understanding of the neurobiology of psychiatric disorders and offer promising avenues for therapeutic intervention, which will be further discussed in the subsequent sections.

### PheWAS Analysis

Utilizing the GWAS ATLAS analysis tool, we aggregated the traits associated with potential causal genes for the three diseases. Given the limited number of anxiety-related sites identified, further selections were challenging. Therefore, we proceeded to conduct PheWAS analysis directly on the identified sites. For patients with depression and schizophrenia, we selected sites through three screening methods for PheWAS analysis (Additional file 2: Tables S6–S9).

**Fig. 5:**
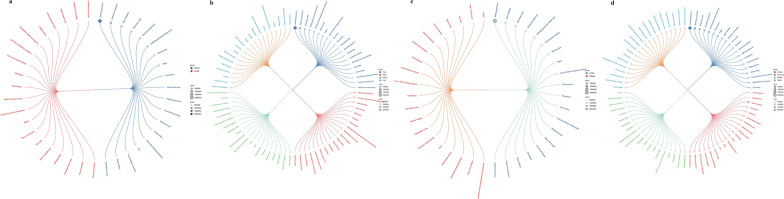
Circular dendrogram displaying the findings of PheWAS analysis. **a** Results of PheWAS for sites related to anxiety; **b** Results of PheWAS for sites related to depression; **c** Results of PheWAS for sites related to schizophrenia; **d** Results of PheWAS on comorbid-related loci for depression and schizophrenia. PheWAS, phenome-Wide Association Studies

Given the multitude of traits associated with each site, we excluded duplicate traits and present only the top 20 primary traits based on the total sample size (Fig. 5). Detailed information on additional traits is available in the Additional Table.

### Drug prediction and molecular docking of candidate compounds

**Fig. 6:**
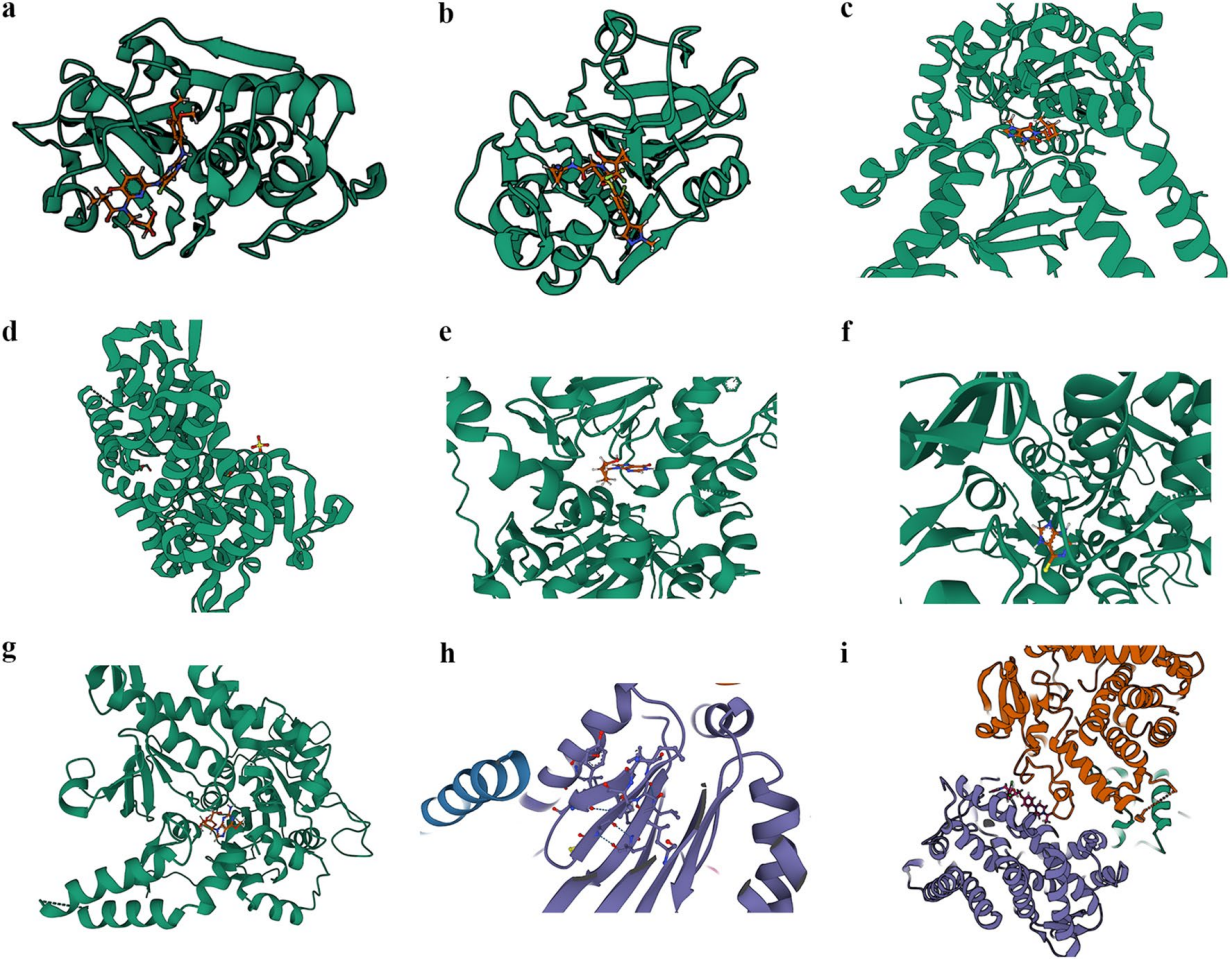
Docking results of available proteins small molecules. **a** CTSS docking Fostamatinib; **b** CTSS docking Petesicatib; **c** NT5C2 docking Pentoxifylline; **d** NT5C2 docking Cytarabine; **e** NT5C2 docking Didanosine; **f** NT5C2 docking Mercaptopurine; **g** NT5C2 docking Nelarabine; **h** PSMA4 docking Cotinine; **i** CYP21A2 docking Ketoconazole. (The PDB number of CTSS is 1GLO, the PDB number of NT5C2 is 2J2C, and the PDB number of CYP21A2 is 5VBU)

Using five databases, including DrugBank, Therapeutic Target Database, ChEMBL, DGIdb, and pharmSnap, we predicted existing drugs for the analyzed and screened sites. We identified a total of 12 potentially effective existing drugs. Excluding zinc ions and copper ions, which were too small for docking, and clozapine, which had well-established efficacy and extensive research, we conducted molecular docking for the remaining drugs with the target proteins using Autodock Vina v.1.2.2. We calculated the binding energies for each interaction (Table 2 and Additional file 2: Table S10) and included docking images illustrating the strongest binding energy for each interaction. The results demonstrate that each candidate drug can bind to the target proteins through visible hydrogen bonds and strong electrostatic interactions (Fig. 6).

**Table 2:** Docking results of available proteins with small molecules

Target	Drug	Binding energy
CTSS	Fostamatinib	− 8.663
CTSS	Petesicatib	− 8.224
NT5C2	Pentoxifylline	− 6.872
NT5C2	Cytarabine	− 6.914
NT5C2	Didanosine	− 6.809
NT5C2	Mercaptopurine	− 5.566
NT5C2	Nelarabine	− 7.717
PSMA4	Cotinine	− 5.703
CYP21A2	Ketoconazole	− 10.552

## Discussion

In this study, we leveraged GWAS data from three large-scale databases to analyze evidence of causal relationships and overlapping genetic structures among anxiety, depression, and schizophrenia. Our findings contribute novel insights into the comorbidity of mental disorders and may offer implications for disease prediction, diagnosis, and treatment.

We observed a significant and consistent genetic correlation between anxiety and depression across multiple databases. Furthermore, LDSC analysis of GWAS data from the Finngen database revealed a weaker genetic correlation between schizophrenia and anxiety and depression, supporting the significant role of genetic factors in these psychiatric disorders.

Mounting evidence suggests that psychiatric disorders share common neurobiological features and exhibit high comorbidity. The p-factor theory posits that a single-dimensional trait can measure an individual's susceptibility to mental disorders, comorbidity among psychiatric disorders, the duration of illnesses, and the severity of symptoms [4]. Various explanations for this high comorbidity include similar life experiences, social stressors [52], environmental factors like trauma [53], and potential medication risks where drugs used to treat one psychiatric disorder may increase the risk of others [54]. Moreover, a recent meta-analysis of studies identified a common brain network sensitive, specific, robust, and consistent with injury-induced effects, mapping the atrophy coordinates of psychiatric disorders to this network [55]. This may reveal shared abnormalities or dysfunctions in certain regions or functions of the brain's neural network, providing stronger evidence for the theory of psychiatric disorders' comorbidity.

Focusing on the comorbidity of anxiety, depression, and schizophrenia, approximately 90% of individuals with depressive disorders exhibit symptoms of anxiety. Patients with comorbid disorders of depression and anxiety can range from 46 to 75% [56], and the comorbidity rate between schizophrenia and anxiety reaches up to 38% [57]. Additionally, the comorbidity rate between schizophrenia and depression is as high as 40% [58]. These data align with our research results. In contrast to traditional observational studies, our LDSC analysis method, which does not require specific familial data, provides broader genomic-level support for the p-factor comorbidity theory.

However, genetic correlation alone cannot distinguish causal relationships from genetic confounding. Therefore, this study conducted a meta-analysis of MR results across three databases, revealing strong causal associations between depression and anxiety, schizophrenia and anxiety, and schizophrenia and depression. Notably, the causal relationship between depression and anxiety, especially Major Depressive Disorder (MDD) and anxiety, has been extensively researched and widely accepted [59]. In contrast, causal studies on schizophrenia and anxiety, as well as schizophrenia and depression, are limited and yield unstable conclusions [60, 61]. Our study results contribute evidence in these aspects. MR analysis outcomes heavily rely on data sources, and by conducting a meta-analysis of MR results from three large-scale databases, we mitigated data biases, obtaining more reliable results. This finding reinforces the notion that individuals with one psychiatric disorder are more prone to developing other psychiatric disorders, emphasizing the necessity of early control for a single psychiatric disorder given the increased difficulty in treating comorbid conditions and the challenging drug selection process.

After confirming the comorbidity and causal relationships among the three psychiatric disorders, this study employed methods such as Three-step SMR Analysis, PPI network analysis, and coloc analysis to explore the causal effects of all circulating proteins and genes in human whole blood on anxiety, depression, and schizophrenia. The goal was to identify disease-specific pathogenic genes and overlapping comorbid genes, providing preliminary clues for drug development. Through coloc analysis, ITIH3 was conclusively associated with the onset of depression, CCS exhibited associations with depression at both the gene and protein levels, and CTSS and DNPH1 were implicated in the onset of schizophrenia at both the gene and protein levels. Shared comorbidity loci between depression and schizophrenia included BTN3A1, PSMB4, and TIMP4.

ITIH3 is a protein involved in the formation of Inter-Alpha-Trypsin Inhibitors (ITIs). Its primary functions include participating in the regulation of inflammation, tissue repair, and inhibiting the activity of proteases. Proteases play a role in processes such as the release, breakdown, and reuptake of neurotransmitters in the nervous system. Abnormal protease activity may disrupt the balance of neurotransmitters, leading to the onset of psychiatric disorders [62]. Additionally, protease abnormalities can affect synaptic plasticity, causing abnormal adaptations in neuronal connections and communication, contributing to the development of psychiatric disorders. Therefore, ITIH3 is intricately involved in the potential occurrence of various psychiatric disorders [63]. Recent research in Japan found a close correlation between ITIH3 polymorphism and prenatal depression symptoms in a case–control study [64]. Genome-wide association studies have also identified ITIH3 loci in a broad depression phenotype, emphasizing its role in the genetic correlation of depression traits and even schizophrenia. The shared genetic risk factors among these disorders suggest common pathogenic pathways [65, 66]. Furthermore, the rs2535629 variant of ITIH3 is associated with the efficacy response to antipsychotic drugs, potentially impacting the treatment of mental health disorders, including depression [67].

CCS protein serves as a companion protein to SOD1, assisting in the correct folding and insertion of copper ions into the SOD1 enzyme. This capability enables CCS to ensure that SOD1 receives necessary copper ions to effectively neutralize reactive oxygen species (ROS), protecting cells from oxidative damage [68]. Additionally, CCS participates in maintaining cellular copper homeostasis, constituting its two major functions. Oxidative stress and copper homeostasis are closely linked to neurodegenerative and psychiatric disorders. The central nervous system is particularly susceptible to oxidative stress, leading to neuronal damage, and such damage may be associated with mental health. Recent research underscores the role of copper in depression, as it is the third most abundant trace metal in the human body after iron and zinc, with high levels of copper accumulation in the brain [69]. The relationship between serum copper and mental disorders is currently debated, with some studies showing a positive correlation between serum copper levels and depression, while others show no correlation or even a negative correlation. However, the widely accepted view is that the imbalance of copper homeostasis leading to oxidative stress and inflammatory responses is associated with depression. In addition to its involvement in copper metabolism, some studies suggest that CCS is also related to zinc ions. CCS may form complexes with zinc ions, playing a crucial regulatory role in psychiatric disorders, including influencing neurotransmitter synthesis and release [70]. Through drug target predictions, we found that copper and zinc ions can bind to CCS, demonstrating their potential as therapeutic drugs, which may represent a novel treatment direction for depression.

CTSS, or Cathepsin S, is a lysosomal cysteine protease enzyme involved in the degradation of proteins within lysosomes. Research highlights Cathepsin S's involvement in memory function in the brain and its association with psychiatric disorders such as MDD, bipolar affective disorder, and schizophrenia [71]. Cathepsin S also plays a role in regulating the integrity of the blood–brain barrier (BBB), and changes in BBB permeability may affect the entry of immune cells and inflammatory factors into the brain, potentially contributing to the onset of psychiatric disorders [72]. Recent genetic studies have found potential associations between the CTSS gene and susceptibility to schizophrenia [73]. However, it's crucial to note that the relationship between CTSS and psychiatric disorders, especially schizophrenia, is complex and multifactorial. Further research is needed to elucidate the specific molecular and cellular processes of lysosomal protease S in psychiatric disorders.

DNPH1 is related to the regulation of axonemal dynein, a motor protein involved in various cellular processes, including retrograde transport in axons and intracellular positioning of organelles. Specific research on the connection between DNPH1 and psychiatric disorders is limited. A recent study on schizophrenia and brain structure found that certain gene variations (SNVs) are associated with schizophrenia and brain features such as surface area and thickness. DNPH1 is highly expressed in the cerebral cortex and is one of the genes significantly associated with schizophrenia and brain structure features [74]. This suggests a potential role of DNPH1 in schizophrenia and its potential as a therapeutic target.

As mentioned earlier, patients with schizophrenia are more likely to experience depression. The overlap in symptoms and genetic risk factors between the two disorders suggests a common etiological mechanism. Factors such as maternal immune activation, social isolation, neurotransmitters, the immune system, environment, and metabolism may contribute to their comorbidity. We have identified shared comorbidity loci between schizophrenia and depression, including BTN3A1, PSMB4, and TIMP4.

BTN3A1, Butyrophilin Subfamily 3 Member A1, belongs to the butyrophilin protein family and encodes a transmembrane protein. Proteins in the butyrophilin family are known to participate in immune system regulation. BTN3A1 plays a crucial role in antigen presentation and is highly expressed in the cerebral cortex [75].This suggests a potential close association between BTN3A1 and psychiatric disorders. However, research on the relationship between BTN3A1 and mental illness is still limited and requires further exploration.

PSMB4 is a proteasome subunit, and the proteasome is a large protein complex responsible for ubiquitin-mediated degradation and recycling of damaged or misfolded proteins to maintain cellular homeostasis. Dysfunction in ubiquitin–proteasome function can lead to the accumulation of misfolded and damaged proteins, contributing to the development of diseases such as Alzheimer's, Parkinson's, and schizophrenia [76, 77]. Impaired proteasome function can also result in mitochondrial dysfunction and oxidative stress, both linked to various mental health conditions. Similarly, the research team led by M-L Wong identified a correlation between certain variations in the PSMB4 gene, revealed through polymorphism analysis of inflammatory genes, and susceptibility to MDD [78]. Therefore, targeting PSMB4 for treatment and thereby improving ubiquitin–proteasome function could be a promising approach to correct psychiatric disorders.

TIMP4's main function is to inhibit metalloproteinases, especially matrix metalloproteinases (MMPs). MMPs are involved in the degradation of various components of the extracellular matrix, such as collagen, gelatin, and proteoglycans [79]. By inhibiting MMPs, TIMP4 regulates tissue remodeling, maintains the extracellular matrix, and participates in synaptic plasticity, neuronal differentiation, and neuroprotection in the central nervous system. One study investigated the correlation between the TIMP gene and protein expression levels and depression.The results indicate that changes in the expression of MMPs and TIMP may be a common factor in recurrent depression and somatic diseases, possibly even serving as a marker [80].

Contrary to expectations, a study in 2013 found no significant correlation between a SNP (rs3755724) encoding TIMP4 and schizophrenia [81]. However, the lack of association with a single SNP does not rule out the potential linkage between TIMP4 and psychiatric disorders. Psychiatric disorders result from complex interactions of multiple genes, environmental factors, and genetic heterogeneity. Other TIMP4 variations or interactions with different genes may still be relevant to psychiatric disorders. Additionally, the study was conducted on a Korean population, and genetic heterogeneity between different populations suggests that the role of TIMP4 in psychiatric disorders may vary across different ethnic groups. Further exploration is needed to understand the relationship between TIMP4 and mental illnesses.

We also observed certain loci that haven't undergone co-localization testing, and we conducted drug predictions and molecular docking for these specific targets. Currently, the evidence for the causal relationship between these loci and the three mental disorders may be relatively limited, requiring further validation.

The strength of this study lies in the fact that, following the determination of the correlation of the three mental disorders through LDSC and MR, we employed SMR and co-localization analysis to estimate the specific causal impact of circulatory proteins and genes on anxiety, depression, and schizophrenia using genetic variation. The SMR design minimizes biases caused by confounding and reverse causation, thus improving causal inference. We conducted analyses in three datasets to ensure the robustness of our findings. Co-localization analysis has been proven as a powerful tool to reveal the pleiotropic effects of certain loci on multiple traits, allowing us to analyze the pathogenic loci and comorbidity mechanisms of the three diseases. Additionally, we used phewas analysis to summarize the correlations and predict related drugs for these loci, providing possibilities for drug development. Another strength is that we primarily limited the analysis to populations of European descent, significantly reducing population stratification bias.

However, some limitations in the analysis of this study should be noted. Firstly, the current literature predominantly addresses the general association between COVID-19 and mental disorders, as well as the overarching trend of genetic susceptibility, rather than delving into specific genetic loci. As a result, this study did not extensively investigate the correlation between COVID-19 patients carrying specific genetic variants and the onset of mental disorders, thereby missing an opportunity to elucidate the significant role of genomic features in mental illness. Despite the exclusion of bias caused by linkage disequilibrium and potential reduction of horizontal pleiotropy through HEIDI tests in co-localization analysis, biases and horizontal pleiotropy cannot be completely eliminated. Furthermore, although the overlap of populations is minimal, there is still some population overlap among the UKBB, Finngen, and PGC datasets, which may introduce bias in the analysis. This could lead to interference effects where experimental conditions may impact each other among certain individuals, disrupting randomization. Additionally, focusing on the analysis primarily on individuals of European descent minimizes population structure bias. However, this may limit the generalizability of our research results to other populations.Secondly, plasma proteomic data is derived from the Icelandic population. However, data on the three mental disorders and the genome are primarily based on European populations. Despite adjusting for the top genetic principal components as population structure indicators in GWAS, differences in the ancestry of data sources may introduce population structure bias. Moreover, it's worth noting that we focused only on the cis-regulatory regions of eQTL and pQTL in our analysis, while trans-regulatory regions could also have a widespread impact on regulatory networks. Finally, further functional experiments are needed to validate the identified loci and predict the efficacy of drugs.

## Conclusions

In this study, we conducted a comprehensive analysis of the genetic relationships and overlapping gene structures among anxiety, depression, and schizophrenia by integrating GWAS data from three large-scale databases. We discovered significant and consistent associations at the genetic level among the three disorders. Through MR analysis, we confirmed relationships among anxiety, depression, and schizophrenia. Furthermore, we explored the associations of circulating proteins and genes with these disorders, identifying specific pathogenic genes that offer preliminary clues for drug development. Despite some limitations, such as the impact of population structure and the need for functional experiments, our findings reinforce the understanding of the genetic mechanisms underlying these psychiatric disorders, providing valuable insights for future personalized treatment and drug development.

### Supplementary Information

**Additional file 1:** Additional Figure S1–S30.

**Additional file 2:** Additional Tables S1–S10.

## Data Availability

GWAS ATLAS is available at atlas.ctglab.nl. The GWAS data, eQTL data, and pQTL data sources are listed in Additional file 2: Table S1. All other data analyzed in this study are available upon reasonable request to the corresponding author.

## Abbreviations

eQTL: Expression quantitative trait loci
FDR: False discovery rate
GWAS: Genome-wide association studies
LDSC: Linkage disequilibrium score regression
MAF: Minor allele frequency
MDD: Major depressive disorder
MMPs: Matrix metalloproteinases
MR: Mendelian randomization
PGC: Psychiatric genomics consortium
PheWAS: Phenome-wide association studies
pQTL: Protein quantitative trait loci
ROS: Reactive oxygen species
SMR: Summary-data-based Mendelian randomization
UKBB: UK biobank
