# Supplementary material for: Dissecting the shared genetic landscape of anxiety, depression, and schizophrenia
Source: J Transl Med. 2024 Apr 18;22:373. doi: 10.1186/s12967-024-05153-3 (PMC11025255; doi:10.1186/s12967-024-05153-3)
Supplement: Supplementary file 1 — Additional file 1: Additional Figure S1–S30. [file 12967_2024_5153_MOESM1_ESM.pdf]

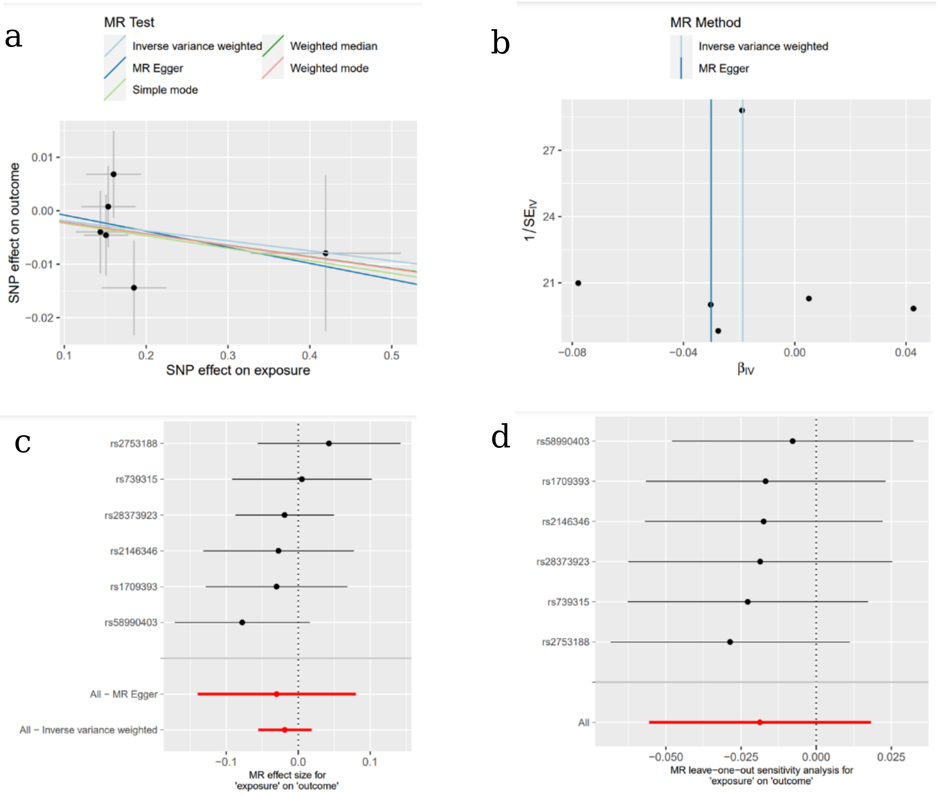

Fig.s1 Results of Mendelian randomization of anxiety(PGC)-depression(finngen). (a) scatter plots of causality; (b) funnel plots; (c)forest plots of each SNPs; (d)leave-one-out plots.

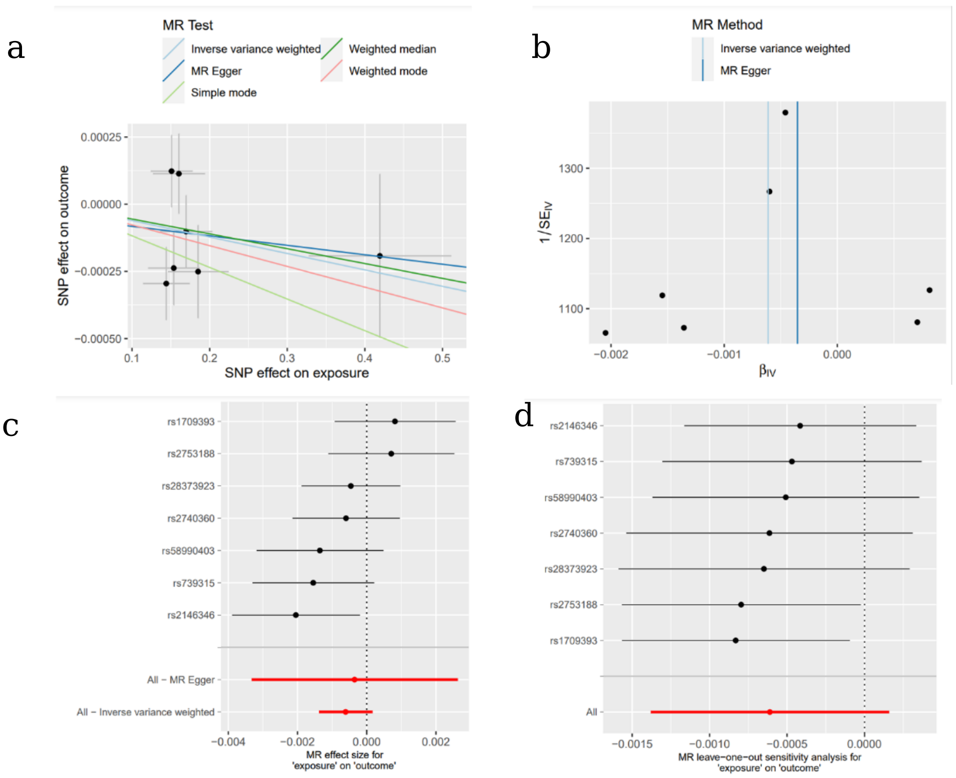

Fig.s2 Results of Mendelian randomization of anxiety(PGC)-depression(UK biobank). (a) scatter plots of causality; (b) funnel plots; (c)forest plots of each SNPs; (d)leave-one-out plots.

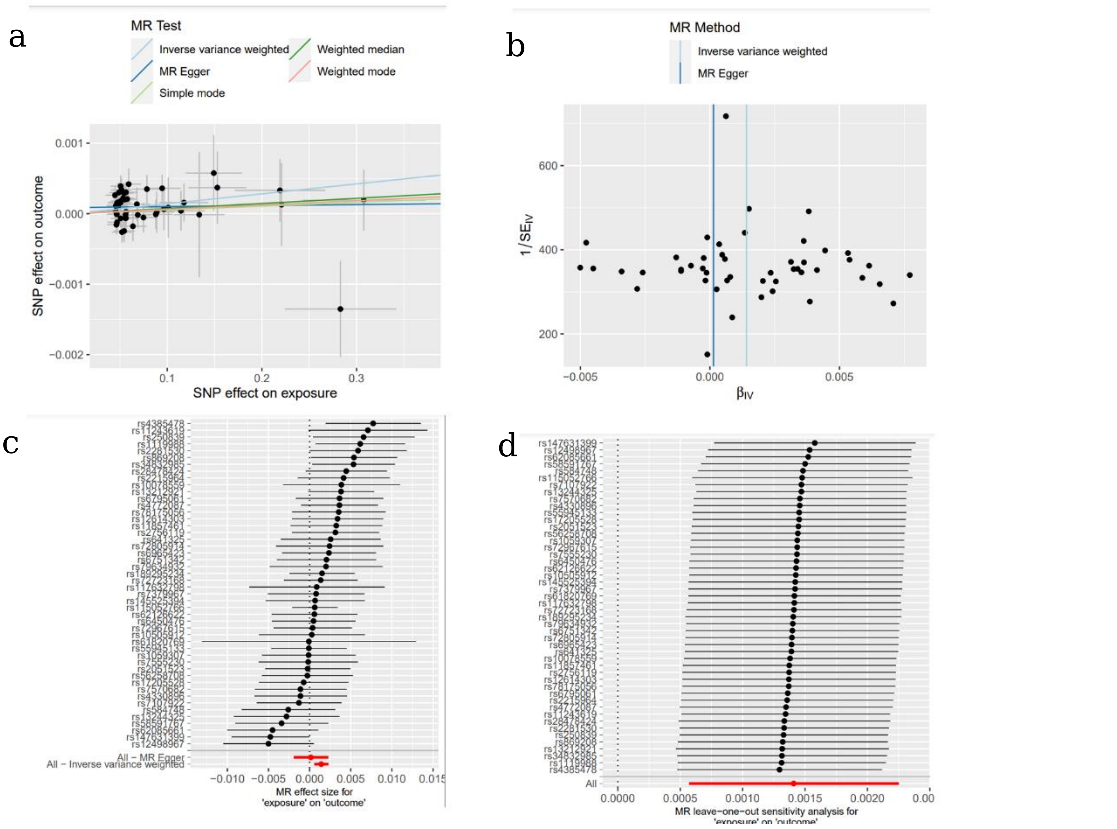

Fig.s3 Results of Mendelian randomization of anxiety(finngen)-depression(UK biobank). (a) scatter plots of causality; (b) funnel plots; (c)forest plots of each SNPs; (d)leave-one-out plots.

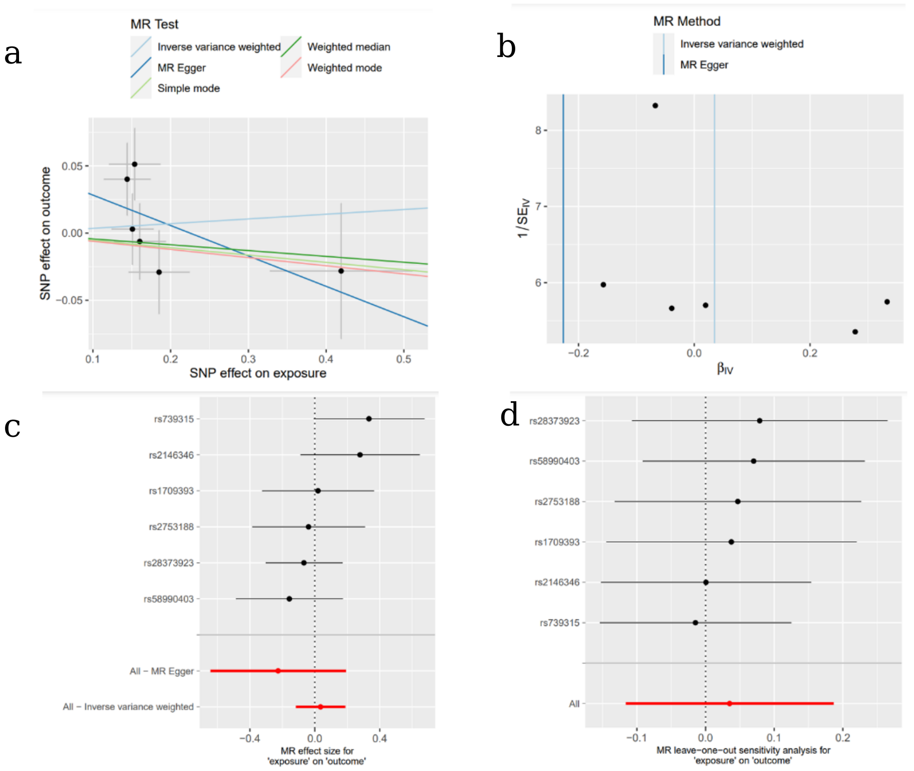

Fig.s4 Results of Mendelian randomization of anxiety(PGC)- schizophrenia (finngen). (a) scatter plots of causality; (b) funnel plots; (c)forest plots of each SNPs; (d)leave-one-out plots.

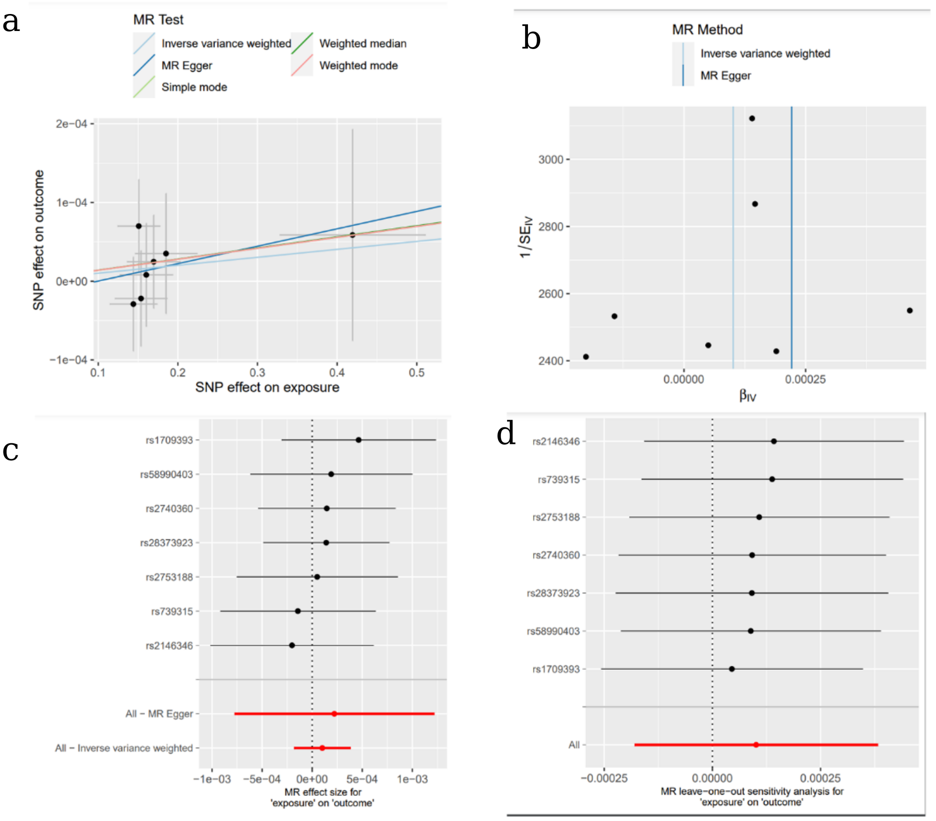

Fig.s5 Results of Mendelian randomization of anxiety(PGC)- schizophrenia (UK biobank). (a) scatter plots of causality; (b) funnel plots; (c)forest plots of each SNPs; (d)leave-one-out plots.

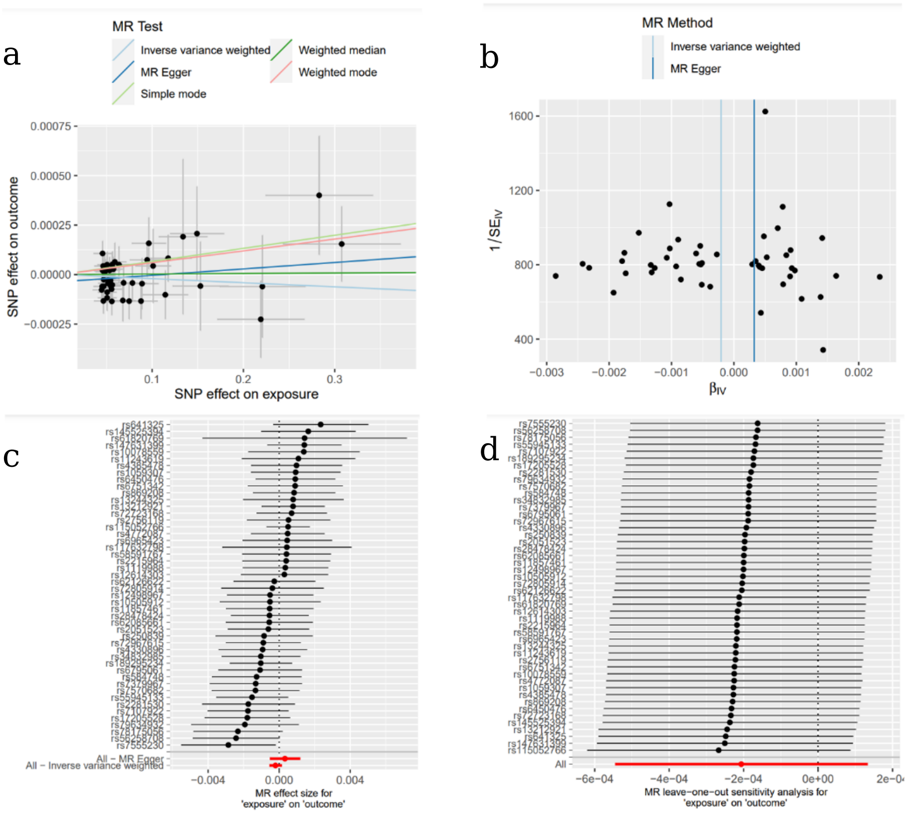

Fig.s6 Results of Mendelian randomization of anxiety(finngen)- schizophrenia (UK biobank)**.** (a) scatter plots of causality; (b) funnel plots; (c)forest plots of each SNPs; (d)leave-one-out plots.

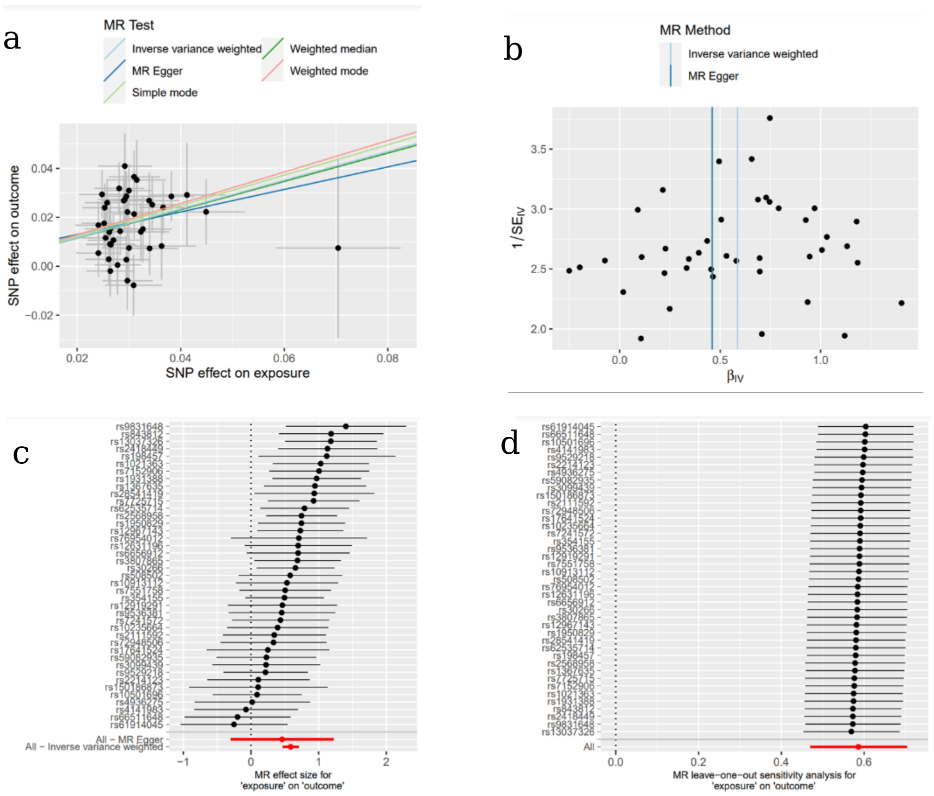

Fig.s7 Results of Mendelian randomization of depression(PGC)- anxiety (finngen). (a) scatter plots of causality; (b) funnel plots; (c)forest plots of each SNPs; (d)leave-one-out plots.

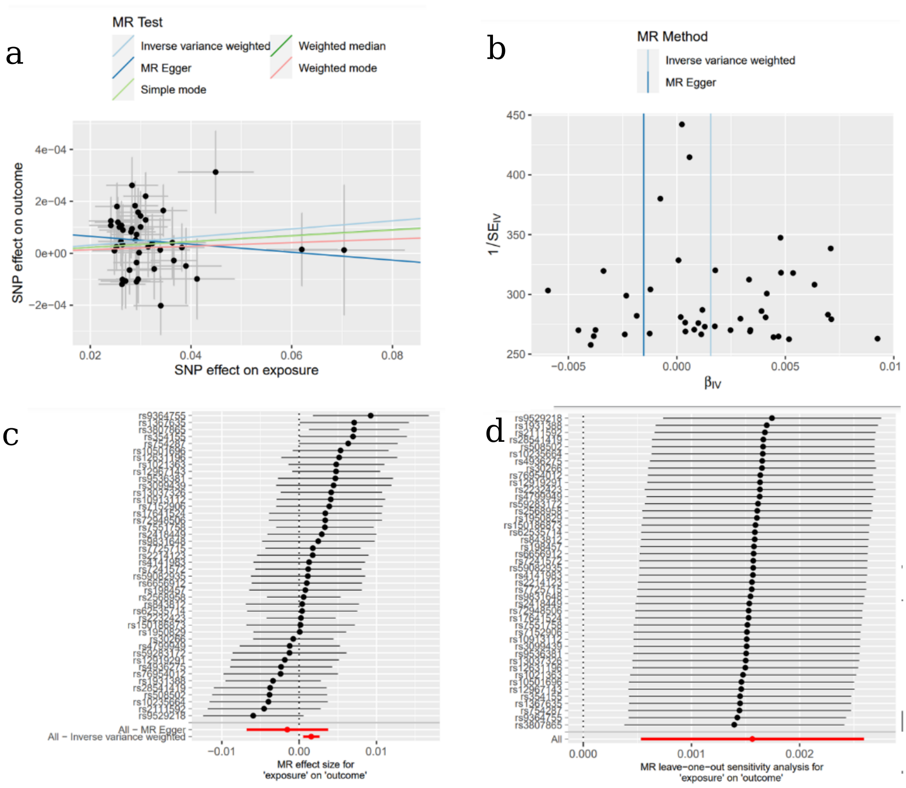

Fig.s8 Results of Mendelian randomization of depression(PGC)- anxiety (UK biobank)**.** (a) scatter plots of causality; (b) funnel plots; (c)forest plots of each SNPs; (d)leave-one-out plots.

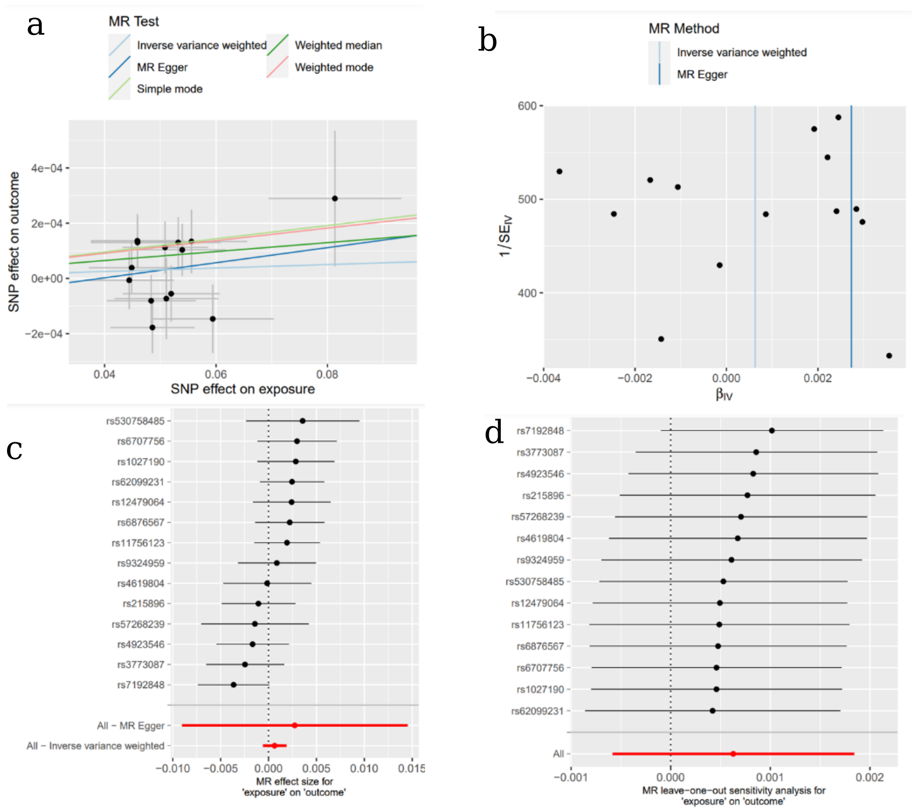

Fig.s9 Results of Mendelian randomization of depression(finngen)-anxiety (UK biobank). (a) scatter plots of causality; (b) funnel plots; (c)forest plots of each SNPs; (d)leave-one-out plots.

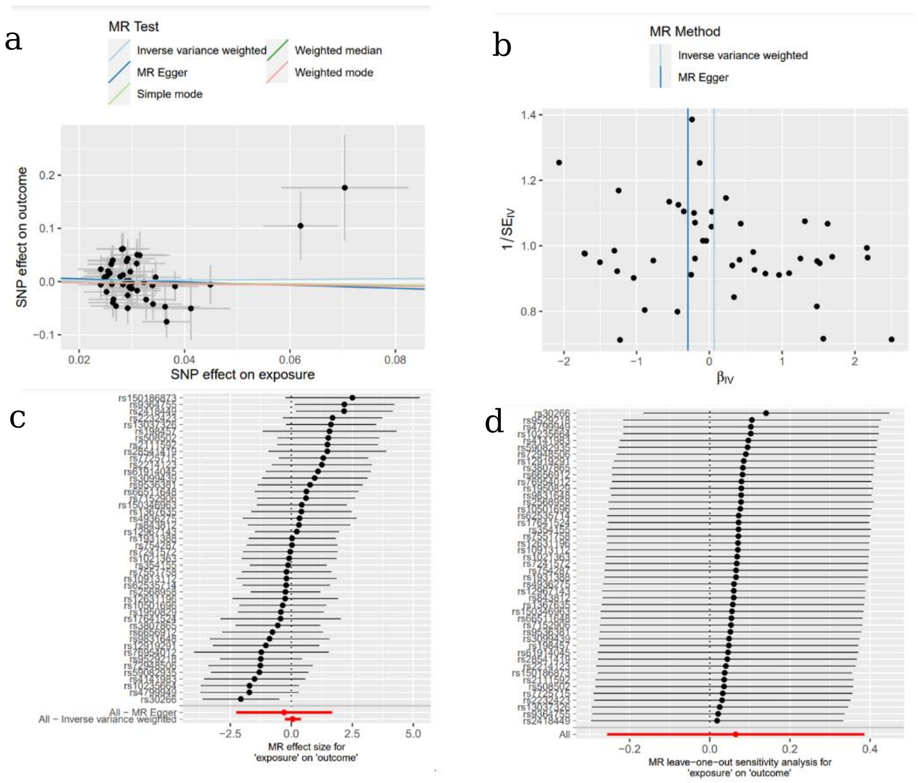

Fig.s10 Results of Mendelian randomization of depression(PGC)-schizophrenia (finngen)**.** (a) scatter plots of causality; (b) funnel plots; (c)forest plots of each SNPs; (d)leave-one-out plots.

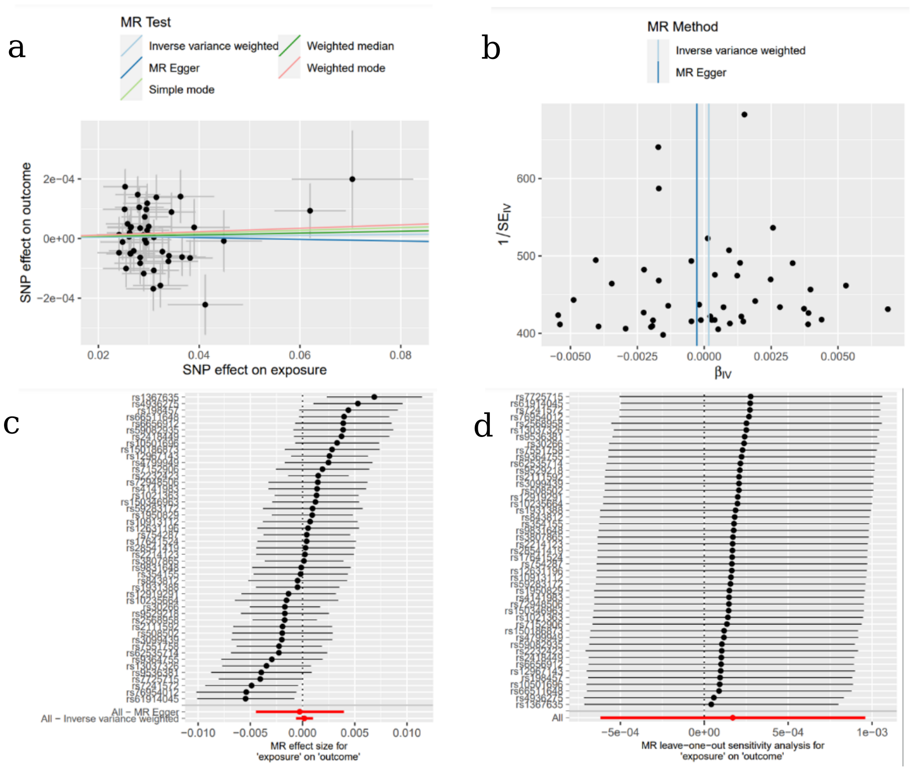

Fig.s11 Results of Mendelian randomization of depression(PGC)-schizophrenia (UK biobank)**.** (a) scatter plots of causality; (b) funnel plots; (c)forest plots of each SNPs; (d)leave-one-out plots.

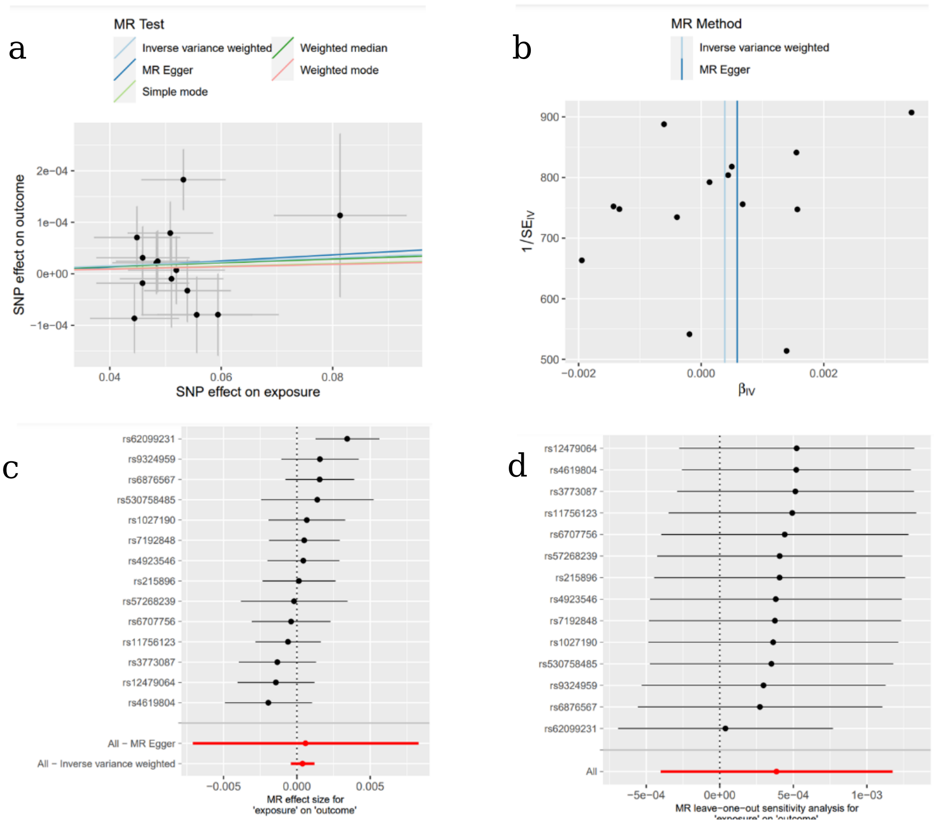

Fig.s12 Results of Mendelian randomization of depression(finngen)-schizophrenia (UK biobank). (a) scatter plots of causality; (b) funnel plots; (c)forest plots of each SNPs; (d)leave-one-out plots.

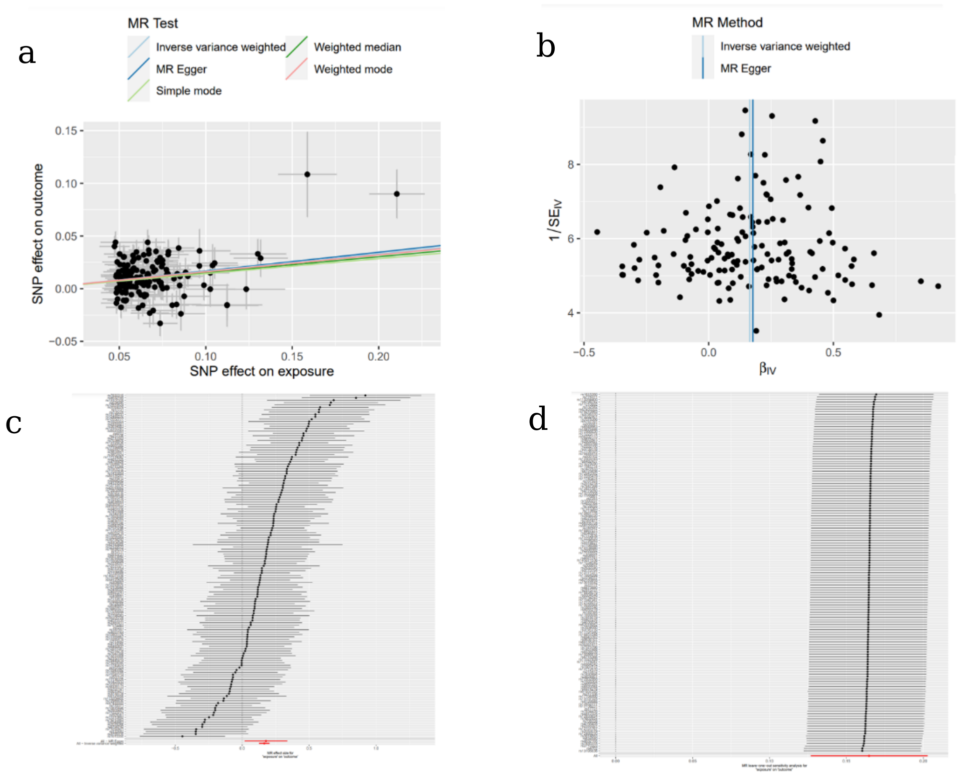

Fig.s13 Results of Mendelian randomization of schizophrenia (PGC)-anxiety (finngen)**.** (a) scatter plots of causality; (b) funnel plots; (c)forest plots of each SNPs; (d)leave-one-out plots.

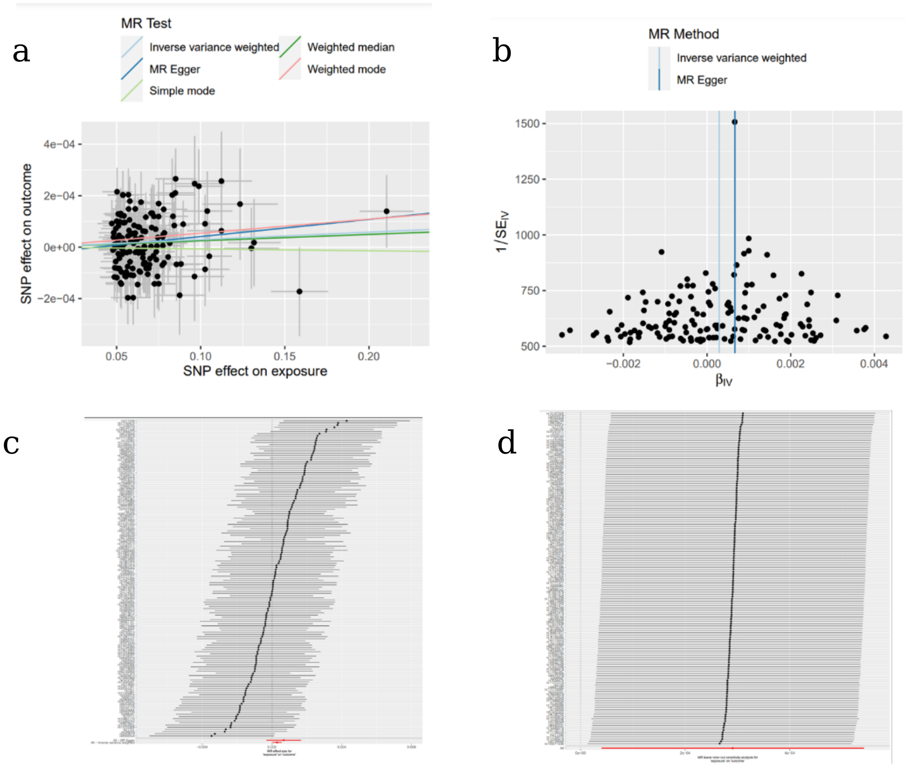

Fig.s14 Results of Mendelian randomization of schizophrenia (PGC)-anxiety (UK biobank). (a) scatter plots of causality; (b) funnel plots; (c)forest plots of each SNPs; (d)leave-one-out plots.

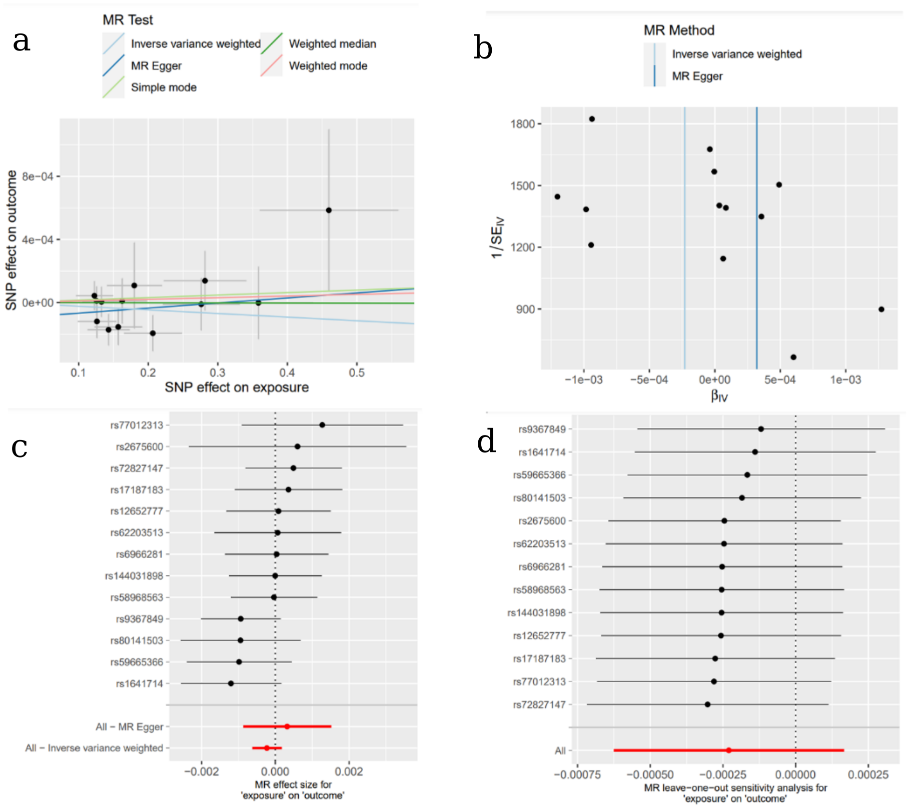

Fig.s15 Results of Mendelian randomization of schizophrenia (finngen)-anxiety (UK biobank)**.** (a) scatter plots of causality; (b) funnel plots; (c)forest plots of each SNPs; (d)leave-one-out plots.

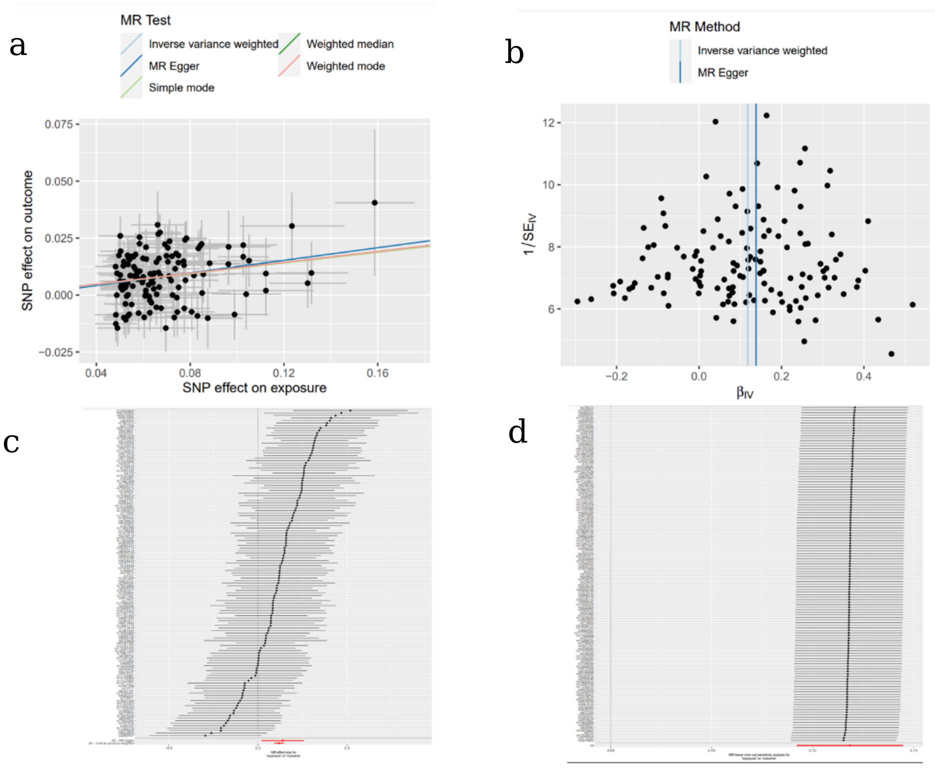

Fig.s16 Results of Mendelian randomization of schizophrenia (PGC)-depression (finngen). (a) scatter plots of causality; (b) funnel plots; (c)forest plots of each SNPs; (d)leave-one-out plots.

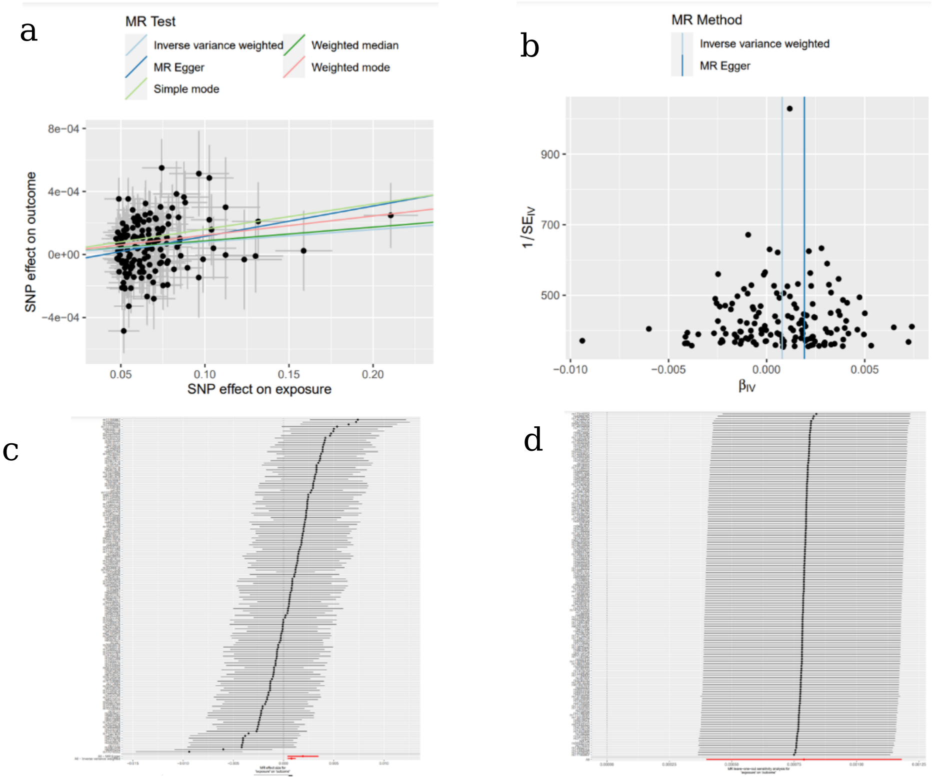

Fig.s17 Results of Mendelian randomization of schizophrenia (PGC)-depression (UK biobank). (a) scatter plots of causality; (b) funnel plots; (c)forest plots of each SNPs; (d)leave-one-out plots.

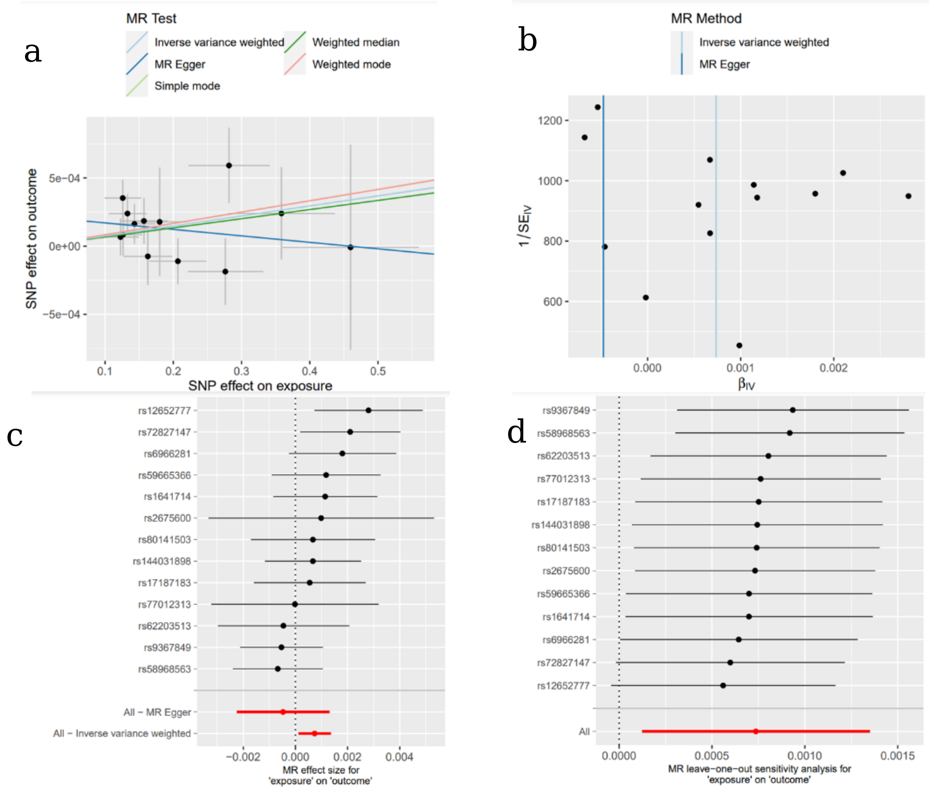

Fig.s18 Results of Mendelian randomization of schizophrenia (finngen)-depression (UK biobank**).** (a) scatter plots of causality; (b) funnel plots; (c)forest plots of each SNPs; (d)leave-one-out plots.

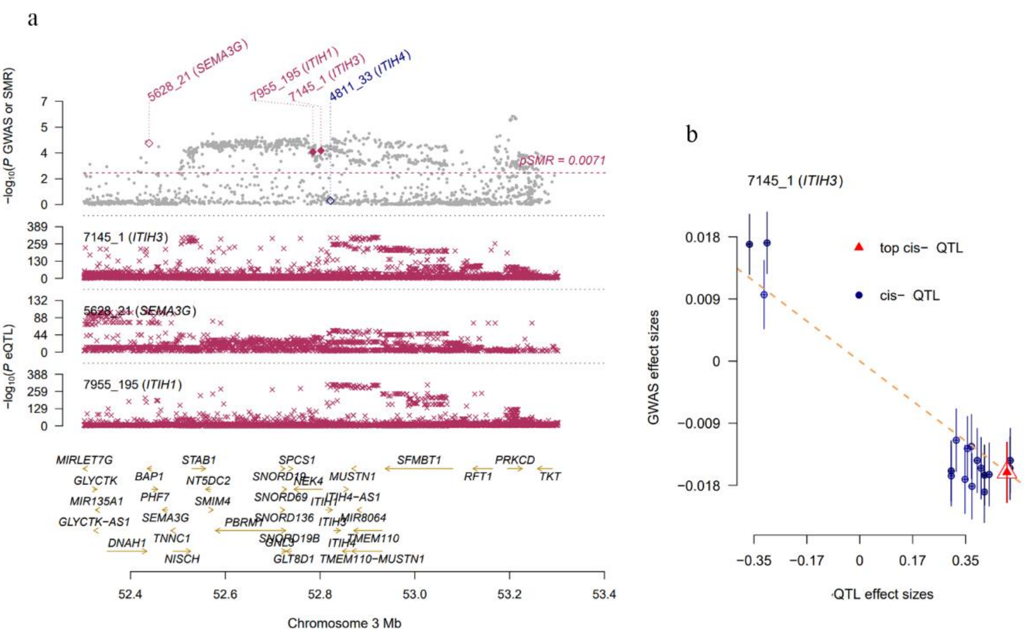

Fig.s19 Depression causation site ITIH3 in SMR analysis.(a) Locus zoom plots showing the consistent protein effects from depression GWAS, and cis-pQTL nearby ITIH3;(b) The scatter plot showed a significant causal relationship between protein expression and the onset of depression.

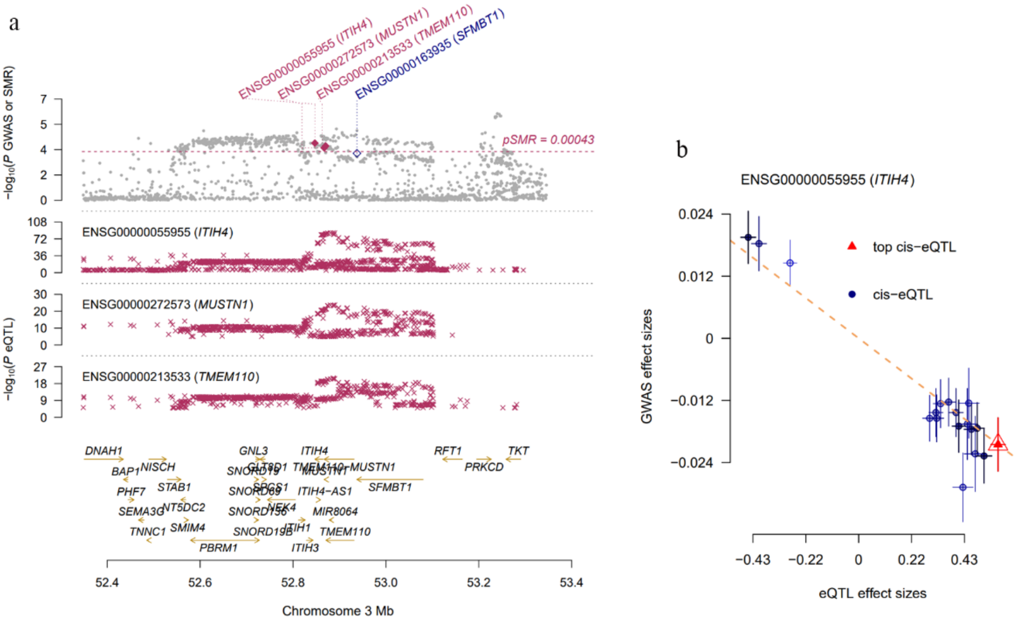

Fig.s20 Depression causation site ITIH4 in SMR analysis.(a) Locus zoom plots showing the consistent genetic effects from depression GWAS, and cis-eQTL nearby ITIH4;(b) The scatter plot showed a significant causal relationship between genetic expression and the onset of depression

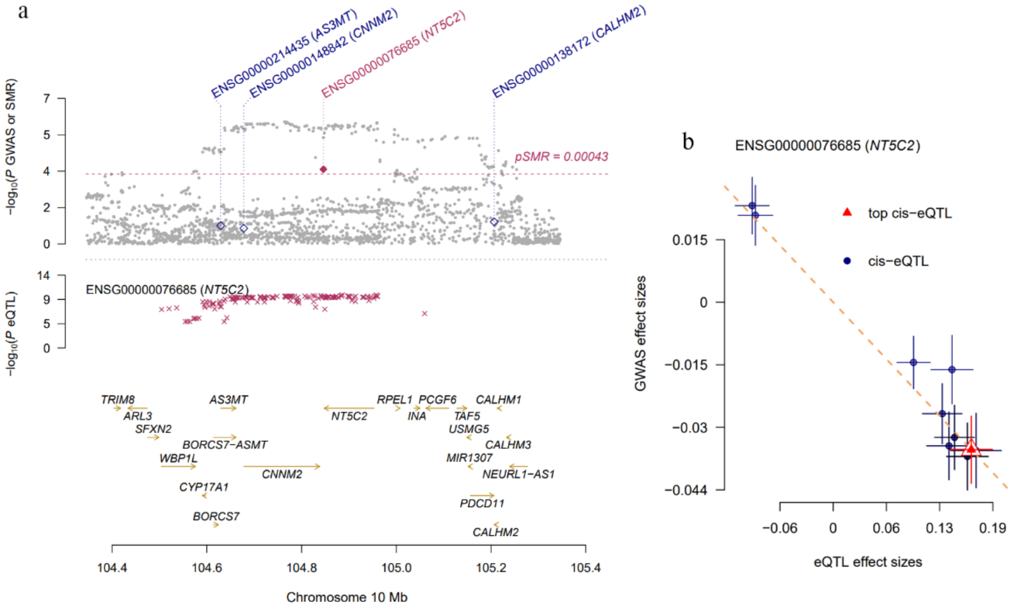

Fig.s21 Depression causation site NT5C2 in SMR analysis.(a) Locus zoom plots showing the consistent genetic effects from depression GWAS, and cis-eQTL nearby NT5C2;(b) The scatter plot showed a significant causal relationship between genetic expression and the onset of depression.

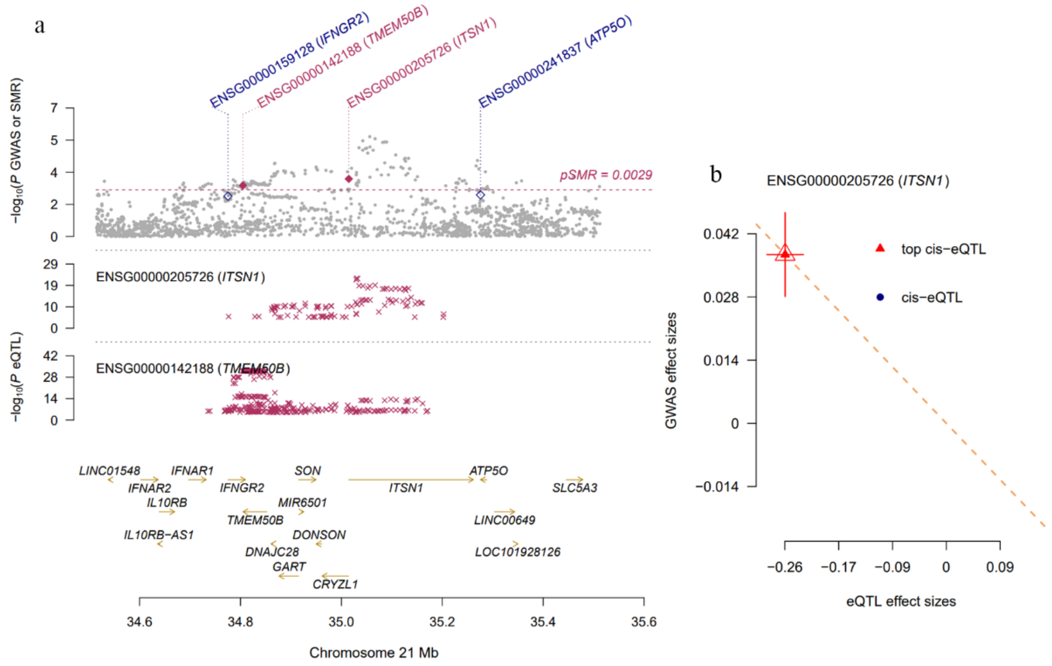

Fig.s22 Schizophrenia causation site ITSN1 in SMR analysis.(a) Locus zoom plots showing the consistent genetic effects from schizophrenia GWAS, and cis-eQTL nearby ITSN1;(b) The scatter plot showed a significant causal relationship between genetic expression and the onset of schizophrenia.

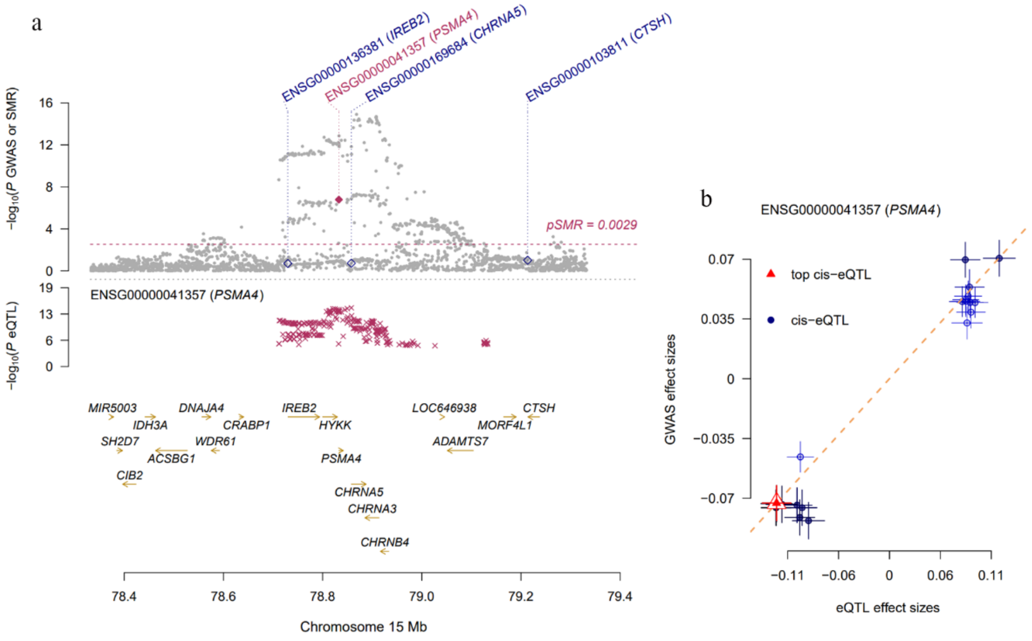

Fig.s23 Schizophrenia causation site PSMA4 in SMR analysis.(a) Locus zoom plots showing the consistent genetic effects from schizophrenia GWAS, and cis-eQTL nearby PSMA4;(b) The scatter plot showed a significant causal relationship between genetic expression and the onset of schizophrenia.

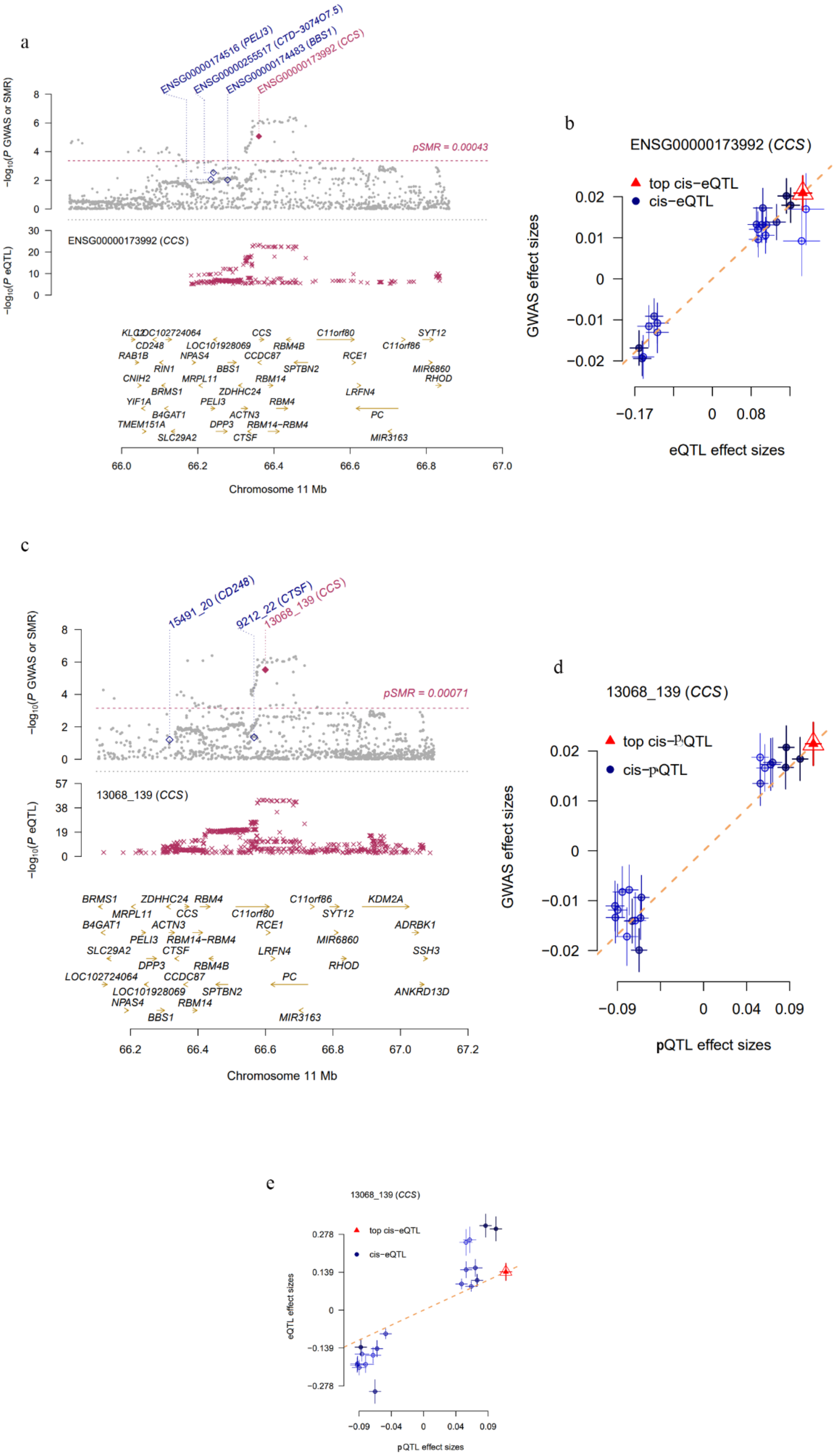

a
ENSG00000174516 (PELI3)
ENSG00000255517 (CTD-3074O7.5)
ENSG00000174483 (BBS1)
ENSG00000173992 (CCS)
pSMR = 0.00043
−log10(P GWAS or SMR)
−log10(P eQTL)
ENSG00000173992 (CCS)
Chromosome 11 Mb
b
ENSG00000173992 (CCS)
top cis−eQTL
cis−eQTL
GWAS effect sizes
eQTL effect sizes
c
15491_20 (CD248)
9212_22 (CTSF)
13068_139 (CCS)
pSMR = 0.00071
−log10(P GWAS or SMR)
−log10(P eQTL)
13068_139 (CCS)
Chromosome 11 Mb
d
13068_139 (CCS)
top cis−pQTL
cis−pQTL
GWAS effect sizes
pQTL effect sizes
e
13068_139 (CCS)
top cis−eQTL
cis−eQTL
eQTL effect sizes
pQTL effect sizes

Fig.s24 Depression causation site CCS in SMR analysis.(a) Locus zoom plots showing the consistent genetic effects from depression GWAS, and cis-eQTL nearby CCS;(b) The scatter plot showed a significant causal relationship between genetic expression and the onset of depression; (c) Locus zoom plots showing the consistent protein effects from depression GWAS, and cis-pQTL nearby CCS;(d) The scatter plot showed a significant causal relationship between protein expression and the onset of depression;(e) The scatter plot showed a significant causal relationship between cis-eQTL and cis-pQTL.

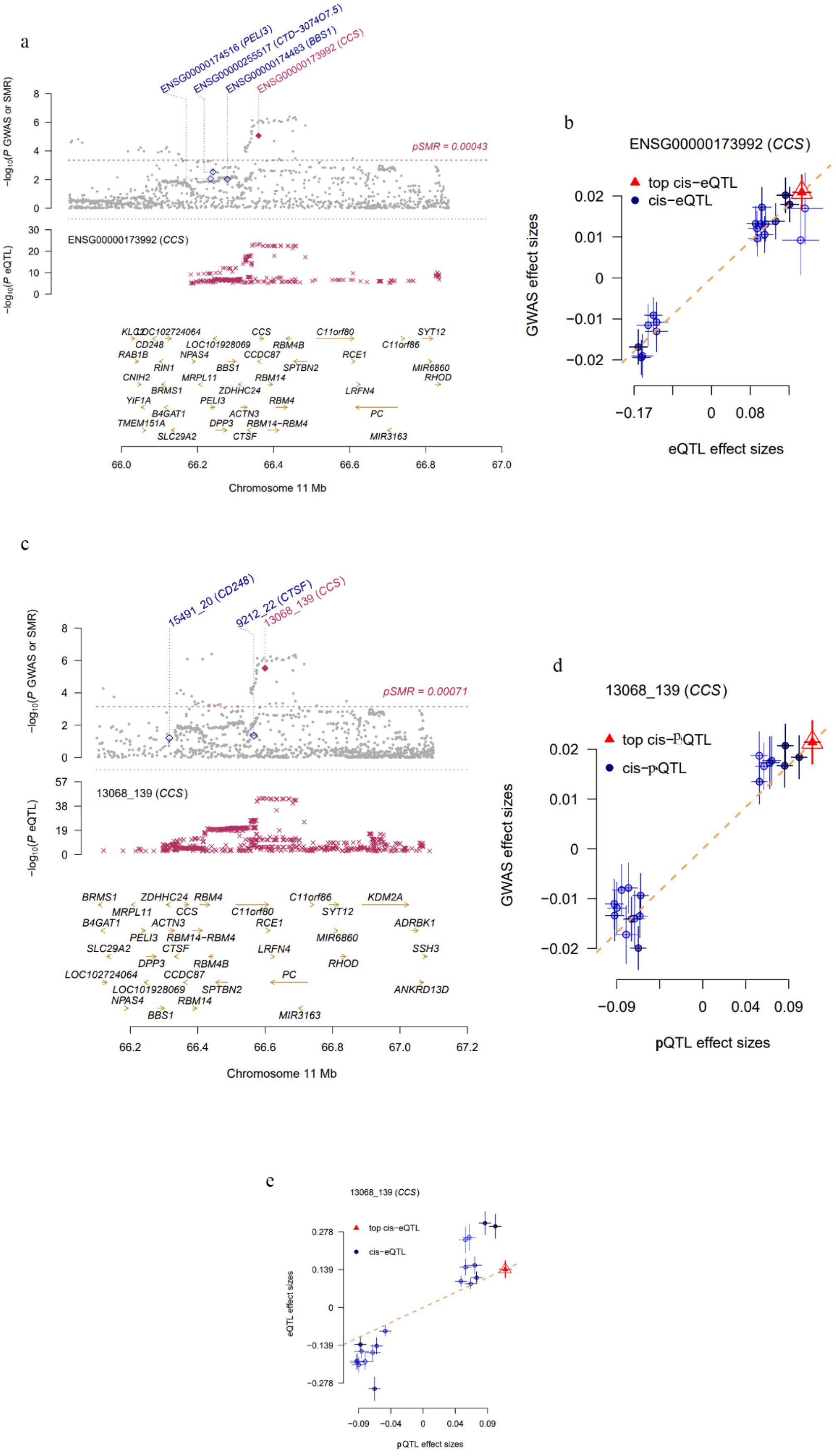

a
ENSG00000174516 (PELI3)
ENSG00000255517 (CTD-3074O7.5)
ENSG00000174483 (BBS1)
ENSG00000173992 (CCS)
−log10(P GWAS or SMR)
pSMR = 0.00043
−log10(P eQTL)
ENSG00000173992 (CCS)
Chromosome 11 Mb
b
ENSG00000173992 (CCS)
top cis−eQTL
cis−eQTL
GWAS effect sizes
eQTL effect sizes
c
15491_20 (CD248)
9212_22 (CTSF)
13068_139 (CCS)
−log10(P GWAS or SMR)
pSMR = 0.00071
−log10(P eQTL)
13068_139 (CCS)
Chromosome 11 Mb
d
13068_139 (CCS)
top cis−pQTL
cis−pQTL
GWAS effect sizes
pQTL effect sizes
e
13068_139 (CCS)
top cis−eQTL
cis−eQTL
eQTL effect sizes
pQTL effect sizes

Fig.s25 Schizophrenia causation site CTSS in SMR analysis.(a) Locus zoom plots showing the consistent genetic effects from schizophrenia GWAS, and cis-eQTL nearby CTSS;(b) The scatter plot showed a significant causal relationship between genetic expression and the onset of schizophrenia; (c) Locus zoom plots showing the consistent protein effects from schizophrenia GWAS, and cis-pQTL nearby CTSS;(d) The scatter plot showed a significant causal relationship between protein expression and the onset of schizophrenia;(e) The scatter plot showed a significant causal relationship between cis-eQTL and cis-pQTL.

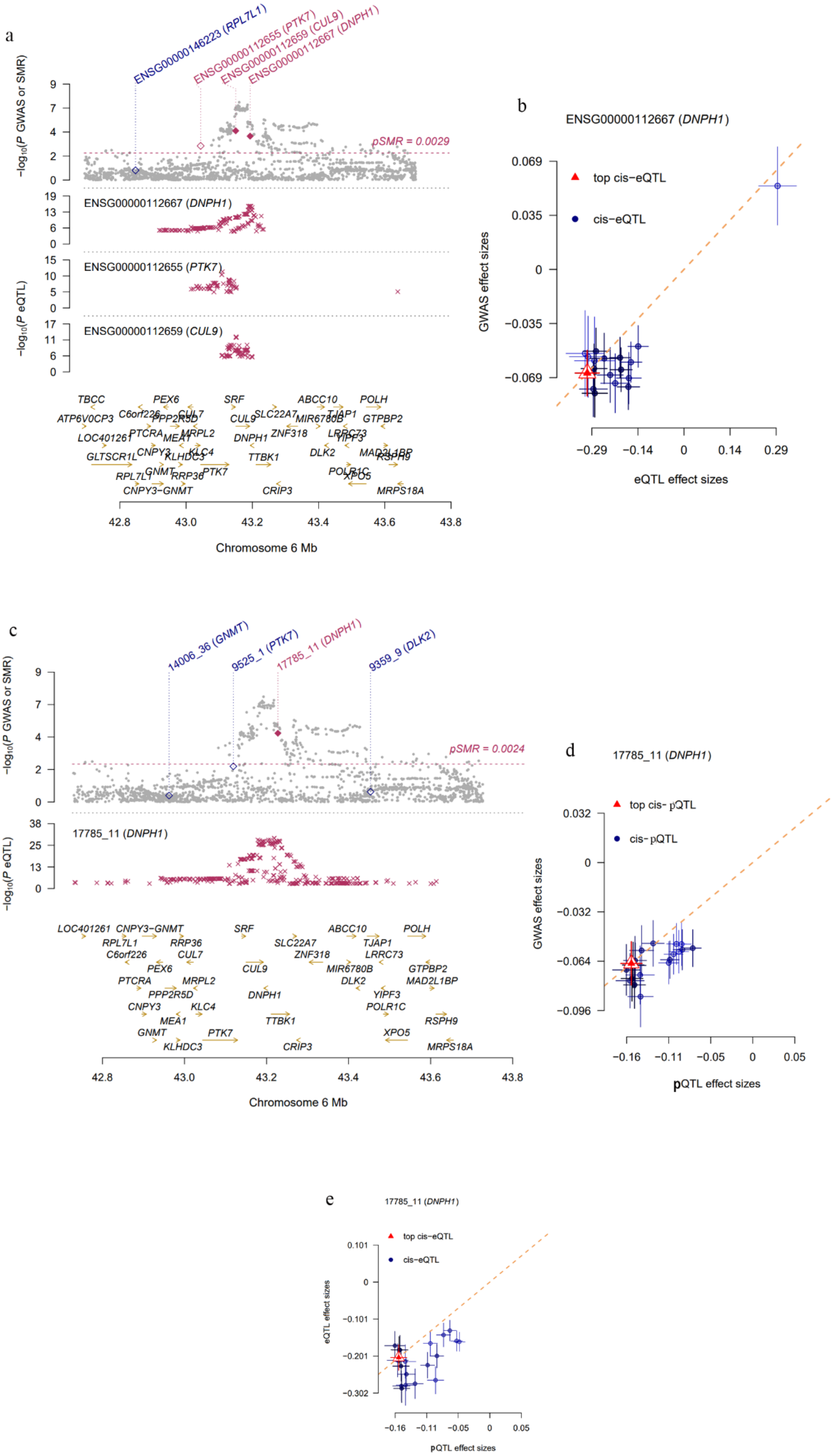
a
ENSG00000146223 (RPL7L1)
ENSG00000112655 (PTK7)
ENSG00000112659 (CUL9)
ENSG00000112667 (DNPH1)
−log10(P GWAS or SMR)
pSMR = 0.0029
ENSG00000112667 (DNPH1)
ENSG00000112655 (PTK7)
ENSG00000112659 (CUL9)
−log10(P eQTL)
TBCC PEX6 SRF ABCC10 POLH
ATP6V0CP3 C6orf226 PPP2R5D CUL7 CUL9 SLC22A7 MIR6780B TJAP1 GTPBP2
LOC401261 PTCRA MEA1 MRPL2 DNPH1 ZNF318 LRRC73 YIPF3
GLTSCR1L CNPY3 KLHDC3 KLC4 TTBK1 DLK2 MAD2L1BP RSPH9
RPL7L1 GNMT RRP36 PTK7 POLR1C XPO5
CNPY3−GNMT CRIP3 MRPS18A
Chromosome 6 Mb
b
ENSG00000112667 (DNPH1)
top cis−eQTL
cis−eQTL
GWAS effect sizes
eQTL effect sizes
c
14006_36 (GNMT)
9525_1 (PTK7)
17785_11 (DNPH1)
9359_9 (DLK2)
−log10(P GWAS or SMR)
pSMR = 0.0024
17785_11 (DNPH1)
−log10(P eQTL)
LOC401261 CNPY3−GNMT SRF ABCC10 POLH
RPL7L1 RRP36 SLC22A7 TJAP1
C6orf226 CUL7 ZNF318 LRRC73
PEX6 CUL9 MIR6780B GTPBP2
PTCRA MRPL2 DLK2 MAD2L1BP
PPP2R5D DNPH1 YIPF3
CNPY3 KLC4 POLR1C
MEA1 TTBK1 RSPH9
GNMT PTK7 XPO5
KLHDC3 CRIP3 MRPS18A
Chromosome 6 Mb
d
17785_11 (DNPH1)
top cis− pQTL
cis− pQTL
GWAS effect sizes
pQTL effect sizes
e
17785_11 (DNPH1)
top cis−eQTL
cis−eQTL
eQTL effect sizes
pQTL effect sizes

Fig.s26 Schizophrenia causation site DNPH1 in SMR analysis.(a) Locus zoom plots showing the consistent genetic effects from schizophrenia GWAS, and cis-eQTL nearby DNPH1;(b) The scatter plot showed a significant causal relationship between genetic expression and the onset of schizophrenia; (c) Locus zoom plots showing the consistent protein effects from schizophrenia GWAS, and cis-pQTL nearby DNPH1;(d) The scatter plot showed a significant causal relationship between protein expression and the onset of schizophrenia;(e) The scatter plot showed a significant causal relationship between cis-eQTL and cis-pQTL.

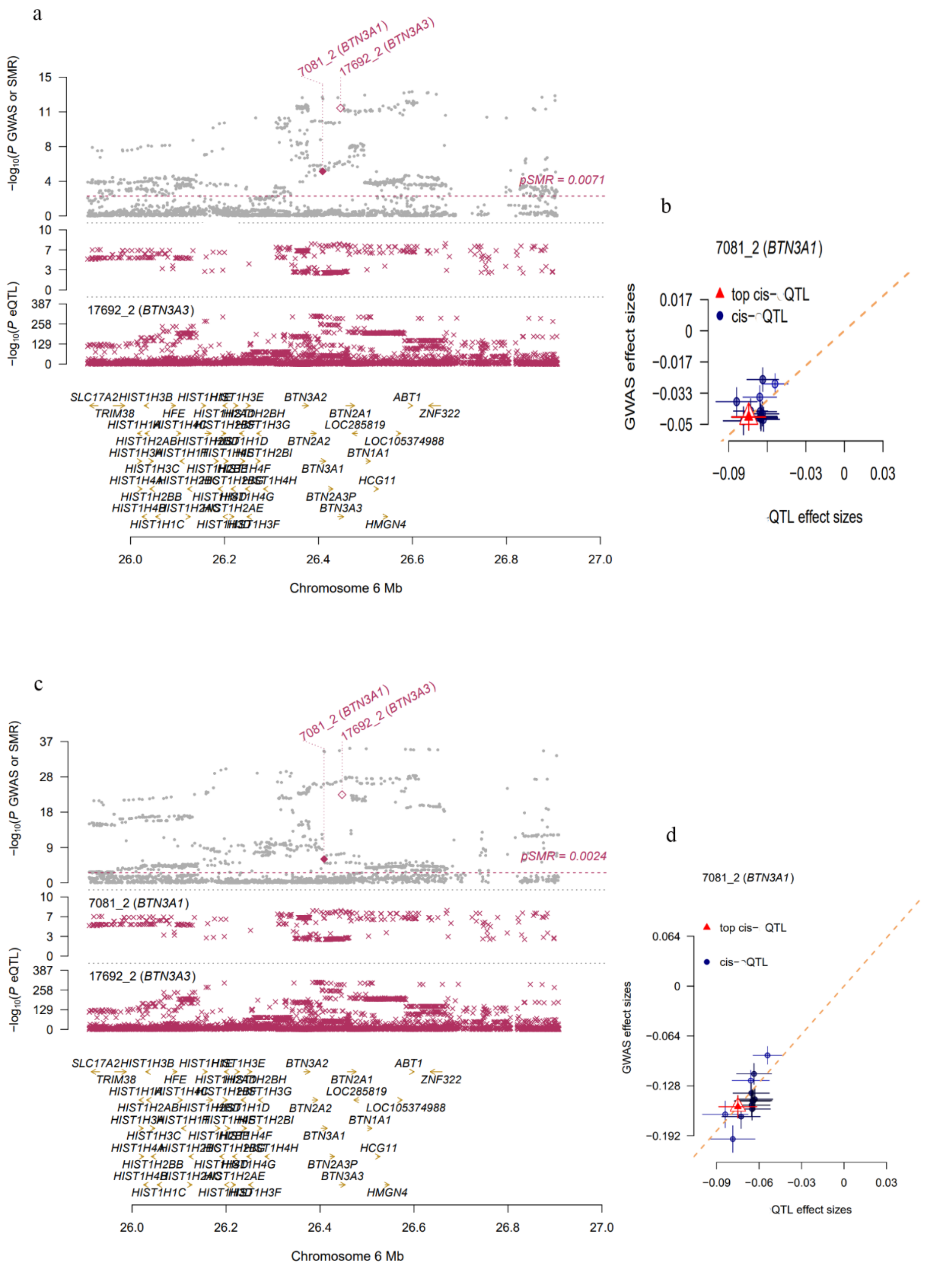

Fig.s27 Depression and schizophrenia causation site BTN3A1 in SMR analysis.(a) Locus zoom plots showing the consistent genetic effects from depression GWAS, and cis-pQTL nearby BTN3A1;(b) The scatter plot showed a significant causal relationship between protein expression and the onset of depression; (c) Locus zoom plots showing the consistent protein effects from

schizophrenia GWAS, and cis-pQTL nearby BTN3A1;(d) The scatter plot showed a significant causal relationship between protein expression and the onset of schizophrenia.

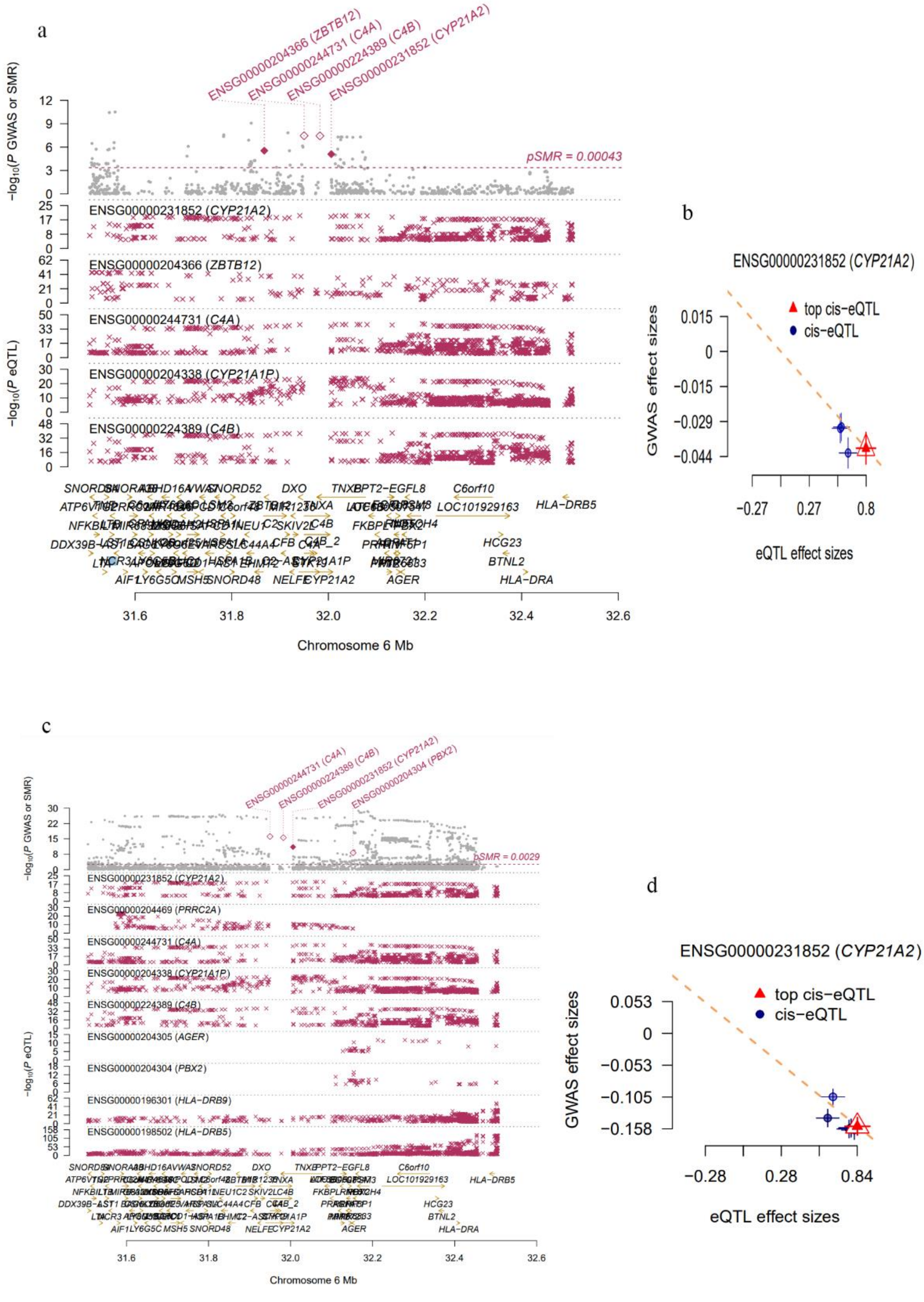

Fig.s28 Depression and schizophrenia causation site CYP21A2 in SMR analysis.(a) Locus zoom

plots showing the consistent genetic effects from depression GWAS, and cis-eQTL nearby CYP21A2;(b) The scatter plot showed a significant causal relationship between genetic expression and the onset of depression; (c) Locus zoom plots showing the consistent genetic effects from schizophrenia GWAS, and cis-eQTL nearby CYP21A2;(d) The scatter plot showed a significant causal relationship between genetic expression and the onset of schizophrenia.

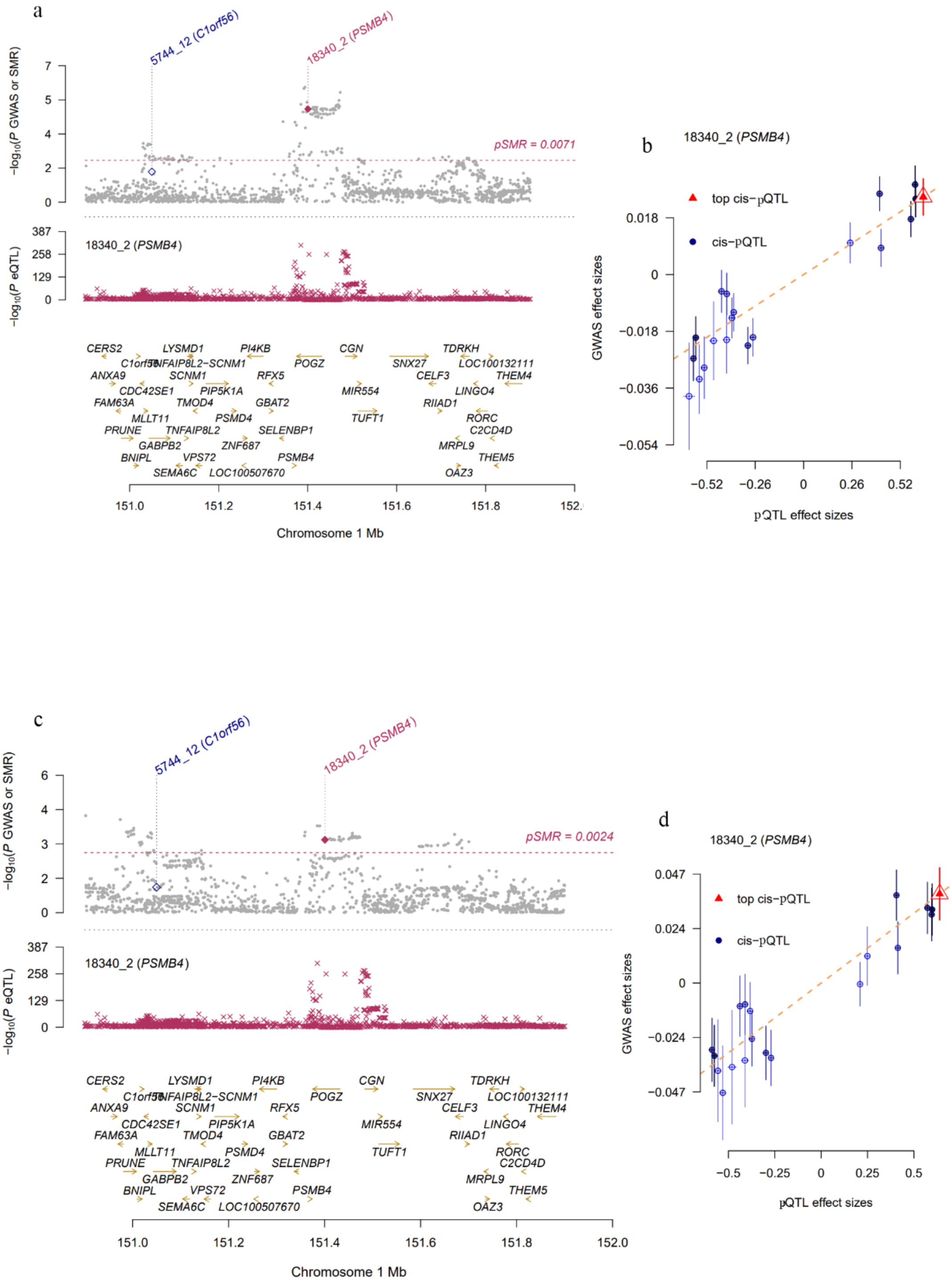

Fig.s29 Depression and schizophrenia causation site PSMB4 in SMR analysis.(a) Locus zoom plots showing the consistent protein effects from depression GWAS, and cis-pQTL nearby PSMB4;(b) The scatter plot showed a significant causal relationship between protein expression and the onset of depression; (c) Locus zoom plots showing the consistent protein effects from schizophrenia

GWAS, and cis-pQTL nearby PSMB4;(d) The scatter plot showed a significant causal relationship between protein expression and the onset of schizophrenia.

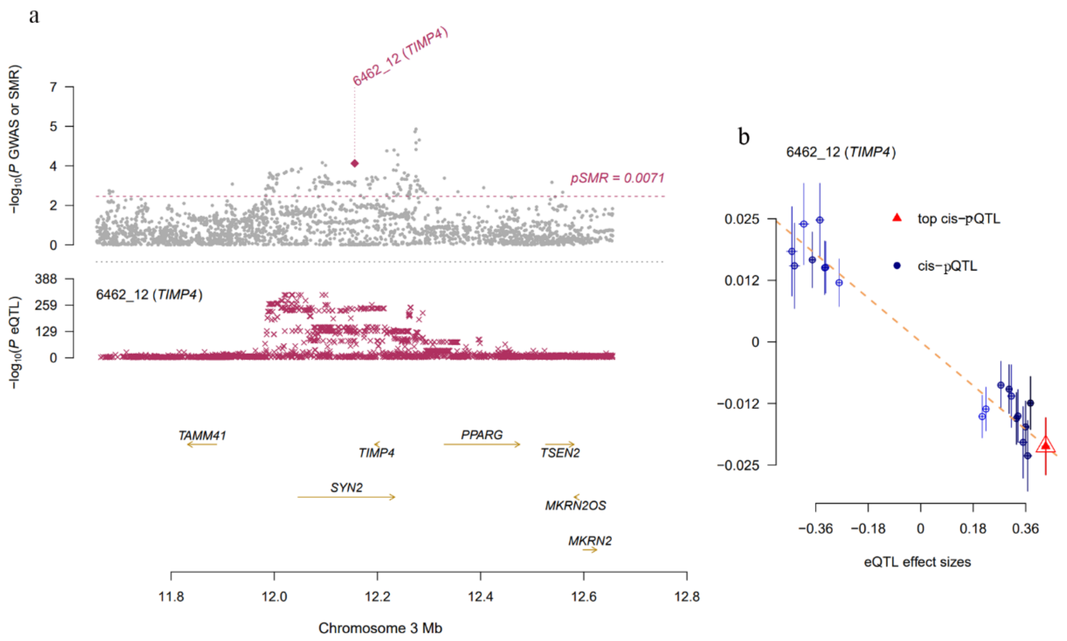

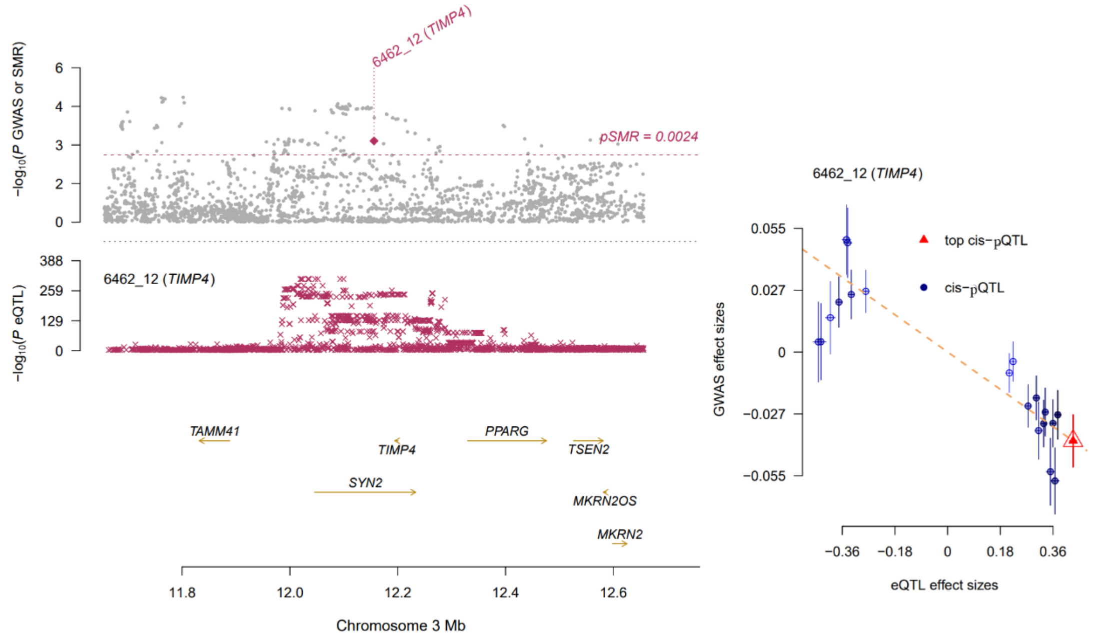

Fig.s30 Depression and schizophrenia causation site TIMP4 in SMR analysis.(a) Locus zoom plots showing the consistent protein effects from depression GWAS, and cis-pQTL nearby TIMP4;(b) The scatter plot showed a significant causal relationship between protein expression and the onset of depression; (c) Locus zoom plots showing the consistent protein effects from schizophrenia GWAS, and cis-pQTL nearby TIMP4;(d) The scatter plot showed a significant causal relationship between protein expression and the onset of schizophrenia.
